# Supplementary material for: Design and implementation of a global site assessment survey among HIV clinics participating in the International epidemiology Databases to Evaluate AIDS (IeDEA) research consortium
Source: PLoS One. 2023 Mar 14;18(3):e0268167. doi: 10.1371/journal.pone.0268167 (PMC10013879; doi:10.1371/journal.pone.0268167)
Supplement: S1 Text — (PDF) [file pone.0268167.s001.pdf]

S1 Text. IeDEA 2020 Site Assessment Survey

The purpose of this survey is to learn about the clinical and support services provided to HIV patients who are enrolled in care at this health facility. This survey is being conducted at all health facilities participating in the International Epidemiology Databases to Evaluate AIDS (IeDEA) network.

This survey is intended to be completed by staff who have in-depth knowledge about the care and services provided to adult and pediatric HIV patients in the HIV clinic or within the health facility or institution. Most questions refer to care and services provided within the HIV clinic. If your health facility does not have a dedicated clinic for HIV care and treatment, please answer for the facility overall, regardless of what unit(s) serves these patients. If your health facility has multiple HIV care and treatment clinics that serve different patient groups, please report on the services provided for adult HIV patients, unless otherwise indicated. A few questions in this survey may require consultation with staff in other units, such as laboratory and pharmacy departments.

The emergence of the COVID-19 pandemic in early 2020 may have resulted in temporary service delivery disruptions and changes in practice. **For Sections 1 – 17 of this survey, please provide information about routine practices and services at your clinic during 2019, prior to the COVID-19 pandemic.** For **Section 18, please provide information about how COVID-19 has affected HIV service delivery**. Remember that there are no incorrect answers to this survey. Your feedback on day-to-day service delivery and routine practices is important for understanding how health facility and service delivery characteristics relate to patient outcomes of interest.

Thank you for your time completing this survey. We are very grateful for your participation.

QUESTIONS	RESPONSES
**1. RESPONDENT INFORMATION**	
1.1 Name of person completing this survey	
1.2 Email address of the person completing the survey	
1.3 Please enter the date this survey is being completed	__ __/__ __/ 2020 (DD / MM / YYYY)
1.4 What is your title?	☐ Head Clinician/Clinical Officer In-Charge<br>☐ Other clinician<br>☐ Site Manager<br>☐ Site Data Manager<br>☐ Head Nurse<br>☐ Other (specify) ____________
**2. PATIENT POPULATION**	
2.1 How would you describe the residence of the population served by this health facility's HIV clinic(s)? *Select one response only*	☐ Predominantly urban<br>☐ Predominantly rural<br>☐ Mixed urban/rural
2.2 What types of patients are served at the HIV clinic(s)? *Check all that apply*	☐ Children (ages 0-9)<br>☐ Adolescents / youth (ages 10-24)<br>☐ Adults – general population (ages 20+)

2.3 In 2019, how often were **specialized or dedicated HIV clinics** held for any of the following patient groups? *Check all that apply. If patient groups are served as part of general clinic population, rather than through dedicated clinics, check "Not Available."*	Available every day the health facility is open	Available on special/ dedicated days	Not available
a. Pediatric patients (ages 0-9)	☐	☐	☐
b. Adolescents/youth (ages 10-24)	☐	☐	☐
c. Pregnant/breast-feeding women	☐	☐	☐
d. Family care clinics	☐	☐	☐
e. Men	☐	☐	☐
f. Patients with comorbidities or opportunistic infections	☐	☐	☐
g. Female sex workers (FSW)	☐	☐	☐
h. Men who have sex with men (MSM)	☐	☐	☐
i. Transgender individuals	☐	☐	☐
j. People with substance use disorders (SUDs)	☐	☐	☐
k. People who inject drugs (PWID)	☐	☐	☐
l. People with mental health disorders (MHDs)	☐	☐	☐
m. Mobile populations	☐	☐	☐
n. Incarcerated populations/prisoners	☐	☐	☐
o. People living with disabilities	☐	☐	☐

QUESTIONS		RESPONSES		
**3. STAFFING & COMMUNITY LINKAGES.** *Please describe staffing situation as it was prior to the COVID-19 pandemic*				
3.1	In 2019, how often were the following categories of staff available at this HIV clinic?	**Available every day clinic is open**	**Available some days**	**Never available**
a.	Pediatrician (general)	☐	☐	☐
b.	Internist, family practitioner, generalist (physician)	☐	☐	☐
c.	Infectious disease or HIV specialist	☐	☐	☐
d.	Mid-level providers (clinical officers, nurses/nurse practitioners, midwives, physician assistants)	☐	☐	☐
e.	Adherence counselors	☐	☐	☐
f.	Peer educators/mentors/navigators	☐	☐	☐
g.	Outreach workers	☐	☐	☐
h.	Nutritionists	☐	☐	☐

3.2	In 2019, how often did this HIV clinic work with any of the following community-based partners to **promote HIV testing**?	Never	Daily/ weekly	Monthly/ quarterly	Less than quarterly
a.	Community health committees, village health teams, or voluntary community-based organizations	☐	☐	☐	☐
b.	Community leaders/officials, community health workers, or community health volunteers	☐	☐	☐	☐
c.	People living with HIV/AIDS (PLWHA) associations or patient support groups	☐	☐	☐	☐
d.	Other associations/support groups (e.g. addiction or mental health support groups, sexual minority support groups, etc.)	☐	☐	☐	☐
e.	Youth groups or peer educator groups	☐	☐	☐	☐
f.	Other (specify)________________________	☐	☐	☐	☐
**3.3**	In 2019, how often did this HIV clinic work with any of the following community-based partners to **trace patients**?	Never	Daily/ weekly	Monthly/ quarterly	Less than quarterly
a.	Community health committees, village health teams, or voluntary community-based organizations	☐	☐	☐	☐
b.	Community leaders/officials, community health workers, or community health volunteers	☐	☐	☐	☐
c.	People living with HIV/AIDS (PLWHA) associations or patient support groups	☐	☐	☐	☐
d.	Other associations/support groups (e.g. addiction or mental health support groups, sexual minority support groups, etc.)	☐	☐	☐	☐
e.	Youth groups or peer educator groups	☐	☐	☐	☐
f.	Other (specify)________________________	☐	☐	☐	☐

**4. HIV TESTING & DIAGNOSIS.** *Please describe practices/service delivery as offered prior to the COVID-19 pandemic*		
4.1	How often were HIV counseling and testing services offered at this health facility?	☐ Services available every day clinic is open<br>☐ Services available some days<br>☐ Services never available **{→ SKIP TO 4.3}**
4.2	What types of HIV testing services were offered at this health facility during 2019?<br>*Check all that apply.*	☐ Opt-out testing (provider-initiated)<br>☐ Opt-in testing (patient-initiated)<br>☐ Partner/couples testing<br>☐ "Family tree" testing (testing of family and other household members)<br>☐ Early infant diagnosis (EID)<br>☐ Rapid HIV tests/Same-day testing<br>☐ HIV self-testing kits<br>☐ Other (specify)________________________
4.3	Which of the following types of HIV testing services were offered <u>in the catchment area</u> of this health facility during 2019?<br>*Check all that apply OR select "None."*	☐ None<br>☐ Voluntary counseling and testing (VCT) at fixed community locations<br>☐ Mobile VCT testing<br>☐ Home testing<br>☐ Self-testing<br>☐ Other (specify)________________________<br>☐ Do not know
4.4	**After a positive result on an HIV screening test at this health facility or elsewhere**, was additional testing done at this health facility to confirm HIV diagnoses prior to initiating patients on ART?	☐ Yes<br>☐ No **{→ SKIP TO 4.5}**<br>☐ Not applicable (All patients initiate ART prior to enrollment at this site) **{→ SKIP TO 4.5}**

QUESTIONS	RESPONSES
4.4a. How was the diagnosis of HIV infection confirmed for adult patients enrolling into care at this site in 2019? *Check all that apply.*	☐ Confirmatory antibody test<br>☐ Confirmation based on HIV viral load (PCR) test<br>☐ Other (specify)____________________
4.4b. How was the diagnosis of HIV infection confirmed for infants <18 months at this site in 2019? *Check all that apply.*	☐ Not applicable (no infants/pediatric patients) **{→ SKIP TO 4.8}**<br>☐ Confirmatory antibody test<br>☐ Confirmation based on HIV DNA or RNA PCR<br>☐ Other (specify)____________________
4.5 In 2019, how often were **early infant diagnosis** (EID) services offered at this health facility? *Check one best response.*	☐ Services available every day clinic is open<br>☐ Services available some days<br>☐ Services never available **{→ SKIP TO 4.8}**
4.6 Was same-day/point of care (POC) DNA PCR EID testing routinely available at this health facility in 2019? ***Routinely available** means that the test could be requested or performed, when needed.*	☐ Yes<br>☐ No
4.7 During 2019, what was the usual turnaround time (in days) for getting early infant diagnosis (EID) test results? ***Turnaround time** means the time from ordering or referring a patient for the test to the time when results are received by the facility/clinic staff.*	___________days<br>☐ EID not available

4.8 During 2019, where were the following **HIV diagnostic tests** typically performed for patients at this HIV clinic? *Confirm whether diagnostic services were provided in the HIV clinic and/or elsewhere at the same health facility, only off-site, or were not available for routine patient care.*	**Provided in HIV Clinic**	**In same health facility (but not at HIV clinic)**	**Only offsite (referral)**	**Not available**
a. HIV-1/HIV-2 antigen/antibody immunoassay test for established HIV infection	☐	☐	☐	☐
b. HIV p24 antigen test for acute HIV infection (i.e., before HIV antibodies are detectable)	☐	☐	☐	☐
c. Virologic assay tests (e.g., HIV RNA, nucleic acid test, nucleic acid amplification test,) for acute HIV infection	☐	☐	☐	☐
d. Supplemental HIV-1/HIV-2 antibody differentiation immunoassay	☐	☐	☐	☐
e. DNA or RNA PCR for early infant diagnosis (EID)	☐	☐	☐	☐

5. CARE FOR NEW PATIENTS (PATIENTS NEWLY TESTING POSITIVE OR TRANSFERRING FROM ANOTHER SITE)

5.1 During 2019, what were the most common entry points into HIV care for patients at this health facility? *Check all that apply.*	☐ Voluntary counseling and testing (VCT) unit<br>☐ Maternal and child health services (e.g. Antenatal care, prevention of mother to child transmission, maternity/labor & delivery, under-5 clinic)<br>☐ Sexually transmitted infection (STI) treatment unit<br>☐ Tuberculosis (TB) unit<br>☐ Outpatient department<br>☐ Inpatient hospitalization<br>☐ Referrals/transfers from other health facilities/sites<br>☐ Other (specify) ____________________________
5.2 During 2019, what type of support services were routinely provided to patients who receive a positive HIV test result at this health facility? *Check all that apply OR select "None." **Routinely** means provided as the standard of care.*	☐ None<br>☐ Psychosocial support from nurse, social worker, counselor, mentor, etc.<br>☐ Partner disclosure counseling and support<br>☐ Referral to support groups<br>☐ Referral to community-based volunteers/workers<br>☐ Other (specify)____________________________________
5.3 During 2019, which of the following screenings were routinely done **at the time of enrollment into HIV care** at this health facility (e.g. newly-diagnosed patients or patients who transfer to this site for HIV care)? *Check all that apply OR select "None." **Routinely** means provided as the standard of care at enrollment.*	☐ None<br>☐ Pregnancy/breastfeeding<br>☐ Testing for latent tuberculosis infection (LTBI)<br>☐ Screening for tuberculosis (TB) disease<br>☐ Sexually-transmitted infection (STI) screening<br>☐ Hepatitis B screening<br>☐ Substance use disorders (alcohol, smoking, illicit drug use, etc.)<br>☐ Mental health disorders (depression, anxiety, post-traumatic stress)
5.4 What types of patients received Cotrimoxazole prophylaxis as the standard of care at the HIV clinic? *Check all that apply OR select "None.".*	☐ None<br>☐ All patients<br>☐ Patients who meet a CD4 threshold<br>☐ Pregnant women<br>☐ Infants/children <18 months<br>☐ Infants/children <5 years<br>☐ Infants/children <10 years<br>☐ TB patients<br>☐ Other (specify) _________________________

QUESTIONS	RESPONSES
5.5 In 2019, was CD4 cell count testing done as the standard of care **prior to ART initiation** (for newly-enrolling patients) or **prior to re-starting ART** (for patients re-entering care at this health facility)?	☐ Yes<br>☐ No
5.6 Where is the laboratory that conducted the majority of the CD4 cell count testing for this HIV clinic in 2019?	☐ Onsite, at the same health facility as the HIV clinic<br>☐ Offsite<br>☐ Not available **{→SKIP TO 5.8}**
5.7 In 2019, were same-day/point of care (POC) CD4 count results routinely available at this health facility?<br>***Routinely available*** *means that the test could be requested or performed, when needed.*	☐ Yes<br>☐ No
5.8 Did this HIV clinic provide care to any pre-ART patients in 2019 (i.e., patients who were enrolled in HIV care but had not initiated ART)?	☐ Yes<br>☐ No **{→ SKIP TO 6.1}**
5.9 What medications were routinely provided to **pre-ART patients** (or routinely prescribed if this clinic does not provide medications directly)?<br>*Check all that apply OR select "None."* ***Routinely*** *means provided (or prescribed) as the standard of care.*	☐ None<br>☐ Isoniazid (or other TB preventive therapies, i.e. 3HP, etc.)<br>☐ Vitamin supplements (i.e., multivitamins)<br>☐ Other (specify) ________________________
5.10 What was done if **pre-ART patients** missed an appointment or did not return for ART services?<br>*Check all that apply OR select "Nothing/No routine follow-up tracing."*	☐ Nothing *(No routine follow-up tracing of pre-ART patients)*<br>☐ Phone call to individual and/or family<br>☐ Send message via letter, email, SMS or online patient portal<br>☐ Home visit by clinic staff or community outreach worker<br>☐ Outreach by peer supporter/mentor<br>☐ Other (specify) ________________
5.11 By December 2019, what were the **criteria for ART initiation** at this health facility?<br>*Select all that apply, or select "Start all patients on ART."*	☐ Start **all** patients on ART regardless of CD4 or clinical criteria<br>☐ Start **some** patients on ART regardless of CD4/clinical criteria (specify)_____________<br>☐ CD4 count ≤500 cells/mm^3 for all or some patients.<br>☐ CD4 count ≤350 cells/mm^3 for all or some patients<br>☐ Other criteria (specify)_______________
5.12 How soon after confirming HIV diagnoses and/or treatment eligibility did patients generally initiate ART in 2019?<br>*Check one best response.*	☐ Same day that ART eligibility is established<br>☐ 1-7 days after establishing ART eligibility<br>☐ 8-14 days after establishing ART eligibility<br>☐ 2-4 weeks after establishing ART eligibility<br>☐ >1 month after establishing ART eligibility
5.13 At this clinic, how many ART readiness counseling sessions were typically conducted before eligible patients initiated ART in 2019?<br>*Check one best response.*	☐ 0 sessions<br>☐ 1 session<br>☐ 2 sessions<br>☐ 3 sessions<br>☐ 4 or more sessions
**6. ART MONITORING, ADHERENCE & RETENTION STRATEGIES.** *Describe practices/service delivery prior to COVID-19*	
6.1 In 2019, what was the standard frequency of refills for patients who are stable on ART?<br>*Check one best response.*	☐ Monthly<br>☐ Every 3 months<br>☐ Every 6 months<br>☐ Other (specify)____________________________________
6.2 In 2019, how was ART medication adherence routinely monitored in patients at this HIV clinic?<br>*Check all that apply OR select "Not applicable."* ***Routinely monitored*** *means monitored as the standard of care.*	☐ Not applicable *(Medication adherence not routinely monitored)*<br>☐ Unstructured assessment of adherence by clinician<br>☐ Structured assessment of adherence by clinician using recall instrument (e.g., recall of missed doses during 24-hour, 3-day, 7-day, 30-day, or other period).<br>☐ Pill counts<br>☐ Pharmacy refills<br>☐ Electronic dose monitoring (MEMS caps)<br>☐ Directly observed treatment<br>☐ Routine viral loads<br>☐ Viral loads for patients suspected of non-adherence.<br>☐ Other (specify)______________________________

QUESTIONS	RESPONSES
**ART MONITORING, ADHERENCE & RETENTION STRATEGIES (CONTINUED)** *Describe practices/service delivery prior to COVID-19*	
6.3 What ART adherence support services were routinely provided to HIV patients at this HIV clinic in 2019?<br>*Check all that apply OR select "None/Not applicable."* ***Routinely provided*** *means provided as the standard of care.*	☐ None/Not applicable<br>☐ One-on-one adherence counseling<br>☐ Group adherence counseling<br>☐ Individual mental health counseling<br>☐ Group mental health counseling<br>☐ Referral to peer support or mentor groups<br>☐ Other (specify) ___________________________
6.4. Which of the following types of adherence aids/reminders were routinely provided to ART patients?<br>*Check all that apply.* ***Routinely provided*** *means provided as the standard of care.*	☐ None/Not applicable<br>☐ Patient education media (written, pictorial, video, etc.)<br>☐ Pill boxes or blister packs<br>☐ Calendars, checklists, or other reminders<br>☐ Alarm clocks, wrist watches, beepers<br>☐ Counseling by pharmacist/pharmacy staff<br>☐ Routine review of medication pick-up<br>☐ Other (specify) ________________________
6.5 In 2019, did this HIV clinic utilize text or voice messaging to support any of the following:<br>*Check all that apply.*	☐ None/Not applicable<br>☐ Adherence to medication<br>☐ Adherence to appointments<br>☐ Follow-up of missed appointments<br>☐ Educational messaging

6.6 During 2019, where were the following **tests** typically performed for patients enrolled in care at this HIV clinic?<br>*Confirm whether diagnostic services are provided in the HIV clinic AND/OR elsewhere at the same health facility, only off-site, or were not available for routine patient care.*	**Provided in HIV Clinic**	**In same health facility (but not at HIV clinic)**	**Only offsite (referral)**	**Not available**
a. Quantitative PCR or HIV viral load assay	☐	☐	☐	☐
b. HIV-1 genotypic drug resistance testing	☐	☐	☐	☐

6.7 In 2019, was same-day/point of care (POC) RNA PCR HIV viral load testing routinely available at this health facility?<br>***Routinely available*** *means that the test could be requested or performed any time it was needed.*	☐ Yes<br>☐ No
6.8 During 2019, what was the usual turnaround time (in days) for getting viral load test results?<br>***Turnaround time*** *means the time from ordering or referring a patient for the test to the time when results are received by the facility/clinic staff.*	___________days<br>☐ Viral load testing not available

7. ROUTINE CARE OF ENROLLED HIV PATIENTS. *Describe practices/service delivery prior to COVID-19*

ROUTINE SCREENING DURING FOLLOW-UP

**7.1. Which of the following <u>screenings</u> were <u>regularly performed during follow-up visits</u> for enrolled HIV patients and where was screening typically conducted?** *Select one best response.*	**Provided in HIV Clinic**	**In same health facility (but not at HIV clinic)**	**Only offsite (referral)**	**Not available**
a. Testing for latent tuberculosis infection (LTBI)	☐	☐	☐	☐
b. Screening for tuberculosis (TB) disease	☐	☐	☐	☐
c. Screening for sexually transmitted infections (STIs)	☐	☐	☐	☐
d. Screening for Hepatitis B virus (HBV)	☐	☐	☐	☐
e. Screening for Hepatitis C virus (HCV)	☐	☐	☐	☐
f. Screening for alcohol and substance use disorders	☐	☐	☐	☐
g. Screening for mental health disorders	☐	☐	☐	☐
h. Cervical cancer screening (visual inspection /PAP smear)	☐	☐	☐	☐
i. Anal PAP screening	☐	☐	☐	☐

COUNSELING SERVICES FOR HIV POSITIVE PATIENTS *Describe practices/service delivery prior to COVID-19*

**7.2 Which of the following <u>counseling services</u> were <u>provided to enrolled HIV patients</u> and where were these services typically provided?** *Select one best response.*	**Provided in HIV Clinic**	**In same health facility (but not at HIV clinic)**	**Only offsite (referral)**	**Not available**
a. Counseling regarding disclosure to sexual partners	☐	☐	☐	☐
b. Education on sexual behavior and safer sex practices	☐	☐	☐	☐
c. Family planning counseling	☐	☐	☐	☐
d. Education on high-risk substance-use behaviors and harm reduction practices	☐	☐	☐	☐

QUESTIONS	RESPONSES			
**OTHER PREVENTIVE AND TREATMENT SERVICES FOR HIV POSITIVE PATIENTS**				
**7.3 During 2019, which of the following preventive and treatment services were provided to enrolled HIV patients and where were these services typically provided?** *Select one best response.*	**Provided in HIV Clinic**	**In same health facility (but not at HIV clinic)**	**Only offsite (referral)**	**Not available**
a. Condoms	☐	☐	☐	☐
b. Pre-exposure prophylaxis (PrEP)	☐	☐	☐	☐
c. Post-exposure prophylaxis (PEP)	☐	☐	☐	☐
d. Voluntary male circumcision services	☐	☐	☐	☐
e. Family planning/contraceptive methods other than condoms	☐	☐	☐	☐
f. Treatment/management of depression	☐	☐	☐	☐
g. Treatment/management of post-traumatic stress disorder (PTSD)	☐	☐	☐	☐
h. Treatment/management of anxiety disorders (other than PTSD)	☐	☐	☐	☐
i. Treatment for alcohol use disorders	☐	☐	☐	☐
j. Treatment for substance abuse disorders (other than alcohol)	☐	☐	☐	☐
k. HPV vaccine	☐	☐	☐	☐
l. Pneumococcal vaccine	☐	☐	☐	☐
m. Hepatitis A vaccine	☐	☐	☐	☐
n. Hepatitis B vaccine	☐	☐	☐	☐
**LABORATORY AND DIAGNOSTIC TESTING SERVICES FOR HIV POSITIVE PATIENTS**				
**7.4** During 2019, where were the following laboratory and diagnostic tests typically performed for patients enrolled in care at this HIV clinic? *Select one best response.*	**Provided in HIV Clinic**	**In same health facility (but not at HIV clinic)**	**Only offsite (referral)**	**Not available**
**Routine laboratory monitoring**<br>a. Complete blood count (e.g., platelets, hematocrit, lymphocytes, hemoglobin)	☐	☐	☐	☐
b. Glucose	☐	☐	☐	☐
c. Creatinine	☐	☐	☐	☐
d. Cholesterol	☐	☐	☐	☐
e. AST (SGOT) and/or ALT (SGPT)	☐	☐	☐	☐
**Infectious disease testing**<br>f. Hepatitis B virus (HBV)	☐	☐	☐	☐
g. Hepatitis C virus (HCV)	☐	☐	☐	☐
h. Syphilis testing (RPR/TPHA/VDRL)	☐	☐	☐	☐
i. STIs other than syphilis	☐	☐	☐	☐
**Other screening & diagnostics**<br>j. Cryptococcal meningitis screening (serum cryptococcal antigen or lateral flow assay)	☐	☐	☐	☐
k. Cryptococcal meningitis diagnosis by CSF India Ink or latex agglutination	☐	☐	☐	☐
l. Ultrasound for liver disease management	☐	☐	☐	☐
**FEES/CHARGES FOR HIV-RELATED CARE AND SERVICES**				
**7.5 During 2019, did HIV patients typically pay any fees (other than insurance co-pays) for the following types of routine and specialized services?** *Select one best response.*	*Please indicate if patients paid fees other than insurance co-pays. Select NA for services not available for routine care.*			
a. Routine clinic visits or consultations	☐ Yes	☐ No	☐ Do not know	☐ NA
b. Specialty clinic visits or consultations	☐ Yes	☐ No	☐ Do not know	☐ NA
c. First line ART regimens	☐ Yes	☐ No	☐ Do not know	☐ NA
d. Second line ART regimens	☐ Yes	☐ No	☐ Do not know	☐ NA
e. TB medications	☐ Yes	☐ No	☐ Do not know	☐ NA
f. Opportunistic infection (OI) medications (e.g. Cotrimoxazole, Bactrim, Septra, TMP-SMX)	☐ Yes	☐ No	☐ Do not know	☐ NA
g. Hepatitis C antiviral medication	☐ Yes	☐ No	☐ Do not know	☐ NA
h. Mental health disorder treatment (e.g. medication, counseling, psychotherapy)	☐ Yes	☐ No	☐ Do not know	☐ NA
i. Psychiatric medications	☐ Yes	☐ No	☐ Do not know	☐ NA
j. Substance use disorder treatment (e.g. medication, counseling, psychotherapy)	☐ Yes	☐ No	☐ Do not know	☐ NA

QUESTIONS	RESPONSES
**7.6 In 2019, did HIV patients typically pay any fee (other than insurance co-pays) for the following laboratory and diagnostic services?** *Select one best response.*	*Please indicate if patients paid fees other than insurance co-pays. Select NA for services not available for routine care.*
**HIV-related tests**	
a. HIV-1/HIV-2 antigen/antibody immunoassay test for established HIV infection	☐ Yes ☐ No ☐ Do not know ☐ NA
b. HIV-1 p24 antigen test for acute HIV-1 infection	☐ Yes ☐ No ☐ Do not know ☐ NA
c. Supplemental HIV-1/HIV-2 antibody differentiation immunoassay	☐ Yes ☐ No ☐ Do not know ☐ NA
d. CD4 testing	☐ Yes ☐ No ☐ Do not know ☐ NA
e. DNA or RNA PCR for early infant diagnosis (EID)	☐ Yes ☐ No ☐ Do not know ☐ NA
f. Quantitative PCR for viral load	☐ Yes ☐ No ☐ Do not know ☐ NA
g. HIV-1 genotypic drug resistance testing	☐ Yes ☐ No ☐ Do not know ☐ NA
**Routine laboratory monitoring**	
h. Complete blood count (e.g., platelets, hematocrit, lymphocytes, hemoglobin etc.)	☐ Yes ☐ No ☐ Do not know ☐ NA
i. Glucose	☐ Yes ☐ No ☐ Do not know ☐ NA
j. Creatinine	☐ Yes ☐ No ☐ Do not know ☐ NA
k. Cholesterol	☐ Yes ☐ No ☐ Do not know ☐ NA
l. AST (SGOT) and/or ALT (SGPT)	☐ Yes ☐ No ☐ Do not know ☐ NA
**Infectious disease testing**	
m. Hepatitis B virus (HBV)	☐ Yes ☐ No ☐ Do not know ☐ NA
n. Hepatitis C virus (HCV)	☐ Yes ☐ No ☐ Do not know ☐ NA
o. Syphilis testing (RPR/TPHA/VDRL)	☐ Yes ☐ No ☐ Do not know ☐ NA
p. STIs other than syphilis	☐ Yes ☐ No ☐ Do not know ☐ NA
**Other screening & diagnostics**	
q. Cryptococcal meningitis screening (serum cryptococcal antigen/lateral flow assay)	☐ Yes ☐ No ☐ Do not know ☐ NA
r. Cryptococcal meningitis diagnosis by CSF India Ink or latex agglutination	☐ Yes ☐ No ☐ Do not know ☐ NA
s. Ultrasound for liver disease management	☐ Yes ☐ No ☐ Do not know ☐ NA
t. Cervical cancer screening	☐ Yes ☐ No ☐ Do not know ☐ NA
u. Anal pap screening	☐ Yes ☐ No ☐ Do not know ☐ NA

8. DIFFERENTIATED HIV CARE (CARE TAILORED TO THE NEEDS OF DIFFERENT PATIENT POPULATIONS) *Describe practices/service delivery prior to COVID-19*

8.1 In 2019, did this health facility offer services during extended hours for HIV patients? *Check all that apply.*	☐ No **{→SKIP TO 8.3}**<br>☐ Services offered during extended opening hours<br>☐ Services offered during weekends
8.2 What types of services were available for HIV patients during extended hours in 2019? *Check all that apply.*	☐ HIV testing & counseling<br>☐ ART adherence counseling<br>☐ ART initiation<br>☐ ART refills<br>☐ General services (Clinical monitoring, check-ups, etc.)<br>☐ Laboratory testing (VL monitoring, CD4 testing, etc.)<br>☐ Other (specify) ____________________
8.3 In 2019, did this HIV clinic provide differentiated care (i.e., care specifically tailored to the needs of different patient populations)?	☐ Yes<br>☐ No **{→SKIP TO 9.1}**
8.4 Which of the following HIV-related services were differentiated (i.e., tailored to the needs of different patient populations) in 2019? *Check all that apply.*	☐ HIV testing<br>☐ ART initiation<br>☐ ART delivery
8.5 Which of the following types of patients were served via **differentiated ART delivery models** at this HIV clinic in 2019? *Check all that apply for differentiated ART delivery, or skip to 9.1.*	☐ None **{→SKIP TO 9.1}**<br>☐ Patients presenting/returning to care with advanced HIV disease (CD4<200 cells/mm^3 and/or WHO clinical stage 4 disease)<br>☐ Patients presenting/returning to care when clinically well<br>☐ Patients clinically stable on ART ("stable patients")<br>☐ Patients on ART with virologic/therapeutic failure ("unstable patients", on ART >1 year)
8.6 Which of the following criteria were used to define patient eligibility for differentiated ART delivery models at this HIV clinic in 2019? *Check all that apply.*	☐ Age thresholds (e.g. minimum or maximum age)<br>☐ Time on ART (e.g. minimum time)<br>☐ CD4 cell count thresholds (e.g., CD4 >500 or <200, etc.)<br>☐ Viral load suppression status<br>☐ Current pregnancy or breast-feeding status<br>☐ Current status of any opportunistic infection (OIs)<br>☐ Patient history of drug reactions or toxicities<br>☐ Other (specify) ____________________

QUESTIONS	RESPONSES	
**DIFFERENTIATED CARE (CONTINUED)** *Describe practices/service delivery prior to COVID-19*		
8.7 In 2019, which of the following types of **differentiated ART delivery models** were offered to eligible patients enrolled in care at this HIV clinic, and when were these models introduced at this clinic?	**Model offered (YES/NO)**	**If model offered, specify year of introduction**
a. Patient managed groups (community ART refill group, community patient-led ART delivery, community adherence group, peer support group, etc.)	☐ Yes ☐ No	☐ YEAR: _______<br>☐ Do not know
b. Healthcare worker managed groups (ART adherence clubs, patient adherence club, youth club, teen club, etc.)	☐ Yes ☐ No	☐ YEAR: _______<br>☐ Do not know
c. Facility-based individual models (fast track, quick pick up, pharmacy refill only without clinical consultation, etc.)	☐ Yes ☐ No	☐ YEAR: _______<br>☐ Do not know
d. Out-of-facility individual models (mobile outreach, fixed community ART distribution points, community pharmacy, home delivery, etc.)	☐ Yes ☐ No	☐ YEAR: _______<br>☐ Do not know
8.8 Is there someone at this HIV clinic who may be contacted for additional information about differentiated HIV care?	☐ No<br>☐ Yes (please provide name and email)<br>Name:______________________________<br>Email:______________________________	

**9. HIV CARE FOR PREGNANT AND POSTPARTUM WOMEN.** *Describe practices/service delivery prior to COVID-19*	
9.1 In 2019, did this health facility provide HIV care and treatment to pregnant women living with HIV?	☐ Yes<br>☐ No **{→SKIP TO 9.4}**
9.2 Where was HIV care provided for **patients who become pregnant while already enrolled in HIV care** at this site? *Check all that apply.*	☐ HIV clinic<br>☐ Antenatal/prenatal clinic<br>☐ Other (specify) ____________________________
9.3 Where did **pregnant women newly diagnosed with HIV during pregnancy initiate ART** at this health facility? *Check all that apply.*	☐ HIV clinic<br>☐ Antenatal/prenatal clinic<br>☐ Other (specify) ____________________________
9.4 In 2019, did this health facility provide HIV care and treatment to **postpartum women** (< 24 months after delivery) living with HIV?	☐ Yes<br>☐ No **{→SKIP TO 10.1}**
9.5 Where was HIV care provided for **postpartum women** (< 24 months after delivery) at this health facility? *Check all that apply.*	☐ HIV clinic<br>☐ Postnatal clinic for postpartum women only<br>☐ Maternal and child health (MCH) clinic for women and infants<br>☐ Other (specify) ____________________________
9.6 Where did **women newly diagnosed with HIV during the postpartum period initiate ART** at this health facility? *Check all that apply.*	☐ HIV clinic<br>☐ Postnatal clinic for postpartum women only<br>☐ Maternal and child health (MCH) clinic for women and infants<br>☐ Other (specify) ____________________________

**10. SERVICES PROVIDED TO PEDIATRIC HIV PATIENTS.** *Describe practices/service delivery prior to COVID-19*	
10.1 In 2019, which of the following services were provided to **pediatric HIV patients** (<10 years) at this health facility? *Check all that apply or select "Not Applicable" if no pediatric patients are served at this health facility.*	☐ Not applicable *(No pediatric patients served at this facility)* **{→SKIP TO 10.5}**<br>☐ Postnatal ARV prophylaxis/prevention of mother-to-child transmission services to HIV-exposed infants<br>☐ ART initiation<br>☐ Infant feeding counseling<br>☐ Male circumcision for infants<br>☐ Immunizations<br>☐ Nutritional support<br>☐ Growth monitoring<br>☐ Integrated Management of Childhood Illness (IMCI)<br>☐ Screening for tuberculosis (TB) disease<br>☐ Testing for latent tuberculosis infection (LTBI)
10.2 In 2019, did this health facility provide HIV care and treatment to infants <24 months of age?	☐ Yes<br>☐ No **{→SKIP TO 10.5}**
10.3 Where was HIV care provided for HIV-exposed infants (< 24 months) at this health facility? *Check all that apply.*	☐ HIV clinic<br>☐ Well-baby clinic (for infants and children only)<br>☐ Maternal and child health (MCH) clinic for women and infants<br>☐ Other (specify) ____________________________
10.4 In 2019, where did infants (<24 months) diagnosed with HIV initiate ART at this health facility? *Check all that apply.*	☐ HIV clinic<br>☐ Well-baby clinic (for infants and children only)<br>☐ Maternal and child health (MCH) clinic for women and infants<br>☐ Other (specify) ____________________________

QUESTIONS	RESPONSES
**PEDIATRIC HIV SERVICES (CONTINUED)**	
10.5. In 2019, did this health facility offer any of the following services for adolescent/youth HIV patients?<br>*Check all that apply or tick "None."*	☐ None *(No dedicated services for adolescent patients)*<br>☐ Dedicated hours or space for youth/adolescent HIV testing & counseling services<br>☐ Dedicated hours or space for youth/adolescent HIV care and treatment services<br>☐ Peer counseling for youth/adolescent HIV patients<br>☐ Support groups specifically for youth/adolescent HIV patients<br>☐ Services to support transition to adult HIV care
**11. ROLL-OUT OF DOLUTEGRAVIR (DTG)-BASED ART REGIMENS.** *Describe current status of DTG roll-out.*	
11.1 Have DTG-based regimens been introduced at this HIV clinic as **first-line ART regimens**?	☐ Yes **{→SKIP TO 11.4}**<br>☐ No
11.2 When do you plan to introduce first-line dolutegravir (DTG)-based regimens?<br>*Check one best response.*	☐ No plans for introducing first-line DTG-based regimens<br>☐ 2020<br>☐ 2021<br>☐ Do not know
11.3 Have any of the following other integrase strand transfer inhibitor (INSTI)-based regimens been introduced as first-line ART regimens at this HIV clinic? *Check all that apply.*	☐ None **{→SKIP TO 11.6}**<br>☐ Elvitegravir (brand name Vitekta) **{→SKIP TO 11.6}**<br>☐ Raltegravir (brand name Isentress) **{→SKIP TO 11.6}**<br>☐ Bictegravir **{→SKIP TO 11.6}**
11.4 When (which month and year) was DTG introduced as a **first-line ART regimen**?	MM / YYYY<br>☐ Do not know
11.5 Currently, which of the following patients are eligible for DTG-based **first-line regimens**?<br>*Check all that apply*	☐ ART-naïve patients<br>☐ Patients with suppressed viral load (as defined locally)<br>☐ Patients with unsuppressed viral load (as defined locally)<br>☐ Patients without known drug resistance<br>☐ Patients with known drug resistance<br>☐ Women not of reproductive age (≥50 years)<br>☐ Women of reproductive age (15-49 years)<br>☐ Pregnant women<br>☐ Men<br>☐ Adolescents<br>☐ Children (specify minimum weight in kg) ___________________<br>☐ Other types of patients (specify)__________________
11.6 Has DTG been introduced as a **2nd-line ART regimen** at this site?	☐ Yes **{→SKIP TO 11.8}**<br>☐ No
11.7 When do you plan to introduce 2nd-line DTG-based regimens?<br>*Check one best response.*	☐ No plans for introducing 2nd-line DTG-based regimens **{→SKIP TO 11.10}**<br>☐ 2020 **{→SKIP TO 11.10}**<br>☐ 2021 **{→SKIP TO 11.10}**<br>☐ Do not know **{→SKIP TO 11.10}**
11.8 When (which month and year) was DTG introduced as a **2nd-line ART** regimen?	MM / YYYY<br>☐ Do not know
11.9 Currently, which of the following patients are eligible for DTG-based **2nd-line ART regimens**?<br>*Check all that apply*	☐ Patients with suppressed viral load (as defined locally)<br>☐ Patients with unsuppressed viral load (as defined locally)<br>☐ Patients without known drug resistance<br>☐ Patients with known drug resistance<br>☐ Women not of reproductive age (≥50 years)<br>☐ Women of reproductive age (15-49 years)<br>☐ Pregnant women<br>☐ Men<br>☐ Adolescents<br>☐ Children (specify minimum weight in kg) ___________________<br>☐ Other types of patients (specify)__________________
11.10 Has DTG been introduced as **a 3rd-line ART regimen** at this site?	☐ Yes **{→SKIP TO 11.12}**<br>☐ No
11.11 When do you plan to introduce **3rd-line DTG-based regimens**?<br>*Check one best response.*	☐ No plans for introducing 3rd-line DTG-based regimens **{→SKIP TO 11.14}**<br>☐ 2020 **{→SKIP TO 11.14}**<br>☐ 2021 **{→SKIP TO 11.14}**<br>☐ Do not know **{→SKIP TO 11.14}**
11.12 When (which month and year) was DTG introduced as a **3rd-line ART regimen**?	MM / YYYY<br>☐ Do not know

QUESTIONS	RESPONSES
**ROLL-OUT OF DTG-BASE REGIMENS (CONTINUED)**	
11.13 Currently, which of the following patients are eligible for DTG-based **3rd-line regimens**? *Check all that apply*	☐ Patients with suppressed viral load (as defined locally)<br>☐ Patients with unsuppressed viral load (as defined locally)<br>☐ Patients without known drug resistance<br>☐ Patients with known drug resistance<br>☐ Women not of reproductive age (≥50 years)<br>☐ Women of reproductive age (15-49 years)<br>☐ Pregnant women<br>☐ Men<br>☐ Adolescents<br>☐ Children (specify minimum weight in kg) ___________________<br>☐ Other types of patients (specify)___________________
11.14 Have DTG-based regimens been rolled out at this HIV clinic as part of a national initiative or an institutional or practice-level initiative? *Select one best response.*	☐ National roll-out of DTG-based regimens<br>☐ Institutional/practice-level roll-out of DTG-based regimens<br>☐ Not applicable (no introduction of DTG-based regimens) **{→SKIP TO 12.1}**
11.15 Is the transition of patients to DTG-based regimens based on viral load monitoring?	☐ Yes<br>☐ No **{→SKIP TO Q11.17}**
11.16 How recent a viral load measure is a patient required to have before transitioning to DTG-based regimens? *Select one best response.*	☐ Viral load measure within previous 6 months<br>☐ Viral load measure within previous 12 months<br>☐ Viral load monitoring criteria varies by patient group
11.17 Is HIV genotypic drug resistance testing performed at the time of switching to DTG-based regimen?	☐ Yes<br>☐ No **{→SKIP TO Q12.1}**
11.18 For which types of patients is HIV genotypic drug resistance testing performed at the time of switching to DTG-based regimen? *Check all that apply*	☐ Adult patients starting on or switching 1st-line DTG-based regimens<br>☐ Adult patients switching to a 2nd-line DTG-based regimen<br>☐ Adult patients switching to a 3rd-line DTG-based regimen<br>☐ Children switching from a PI to a DTG-based regimen<br>☐ Children switching from a NNRTI to a DTG-based regimen<br>☐ Other (specify)_____________________________

**12. TB SCREENING, DIAGNOSIS AND PREVENTIVE THERAPY.** *Describe practices/service delivery prior to COVID-19*	
12.1 Did this HIV clinic have a **TB disease screening algorithm** for adult and/or pediatric patients in 2019?	☐ Yes<br>☐ No **{→SKIP TO 12.4}**
12.2 For each of the following symptoms, please indicate whether it was included in the **TB disease screening algorithm** at this HIV clinic for adult patients, pediatric patients, adult and pediatric patients, or for none.	**Symptom included in TB screening algorithm for,,,,**
a. Cough	☐ Adults ☐ Children ☐ Adults & children ☐ None
b. Fever	☐ Adults ☐ Children ☐ Adults & children ☐ None
c. Night sweats	☐ Adults ☐ Children ☐ Adults & children ☐ None
d. Weight loss	☐ Adults ☐ Children ☐ Adults & children ☐ None
e. History of contact with a case of TB	☐ Adults ☐ Children ☐ Adults & children ☐ None
f. Poor weight gain/failure to thrive	☐ Children ☐ None
g. Fatigue/decreased playfulness	☐ Children ☐ None
h. Other (Specify)____	☐ Adults ☐ Children ☐ Adults & children ☐ None

12.3 For what ages are these screening algorithms used? *Please provide **minimum age for adult algorithm** and **maximum age for child algorithm***	**Adult TB screening algorithm**<br>☐ Minimum age_____ (years)<br>☐ NA (no adult patients)	**Child TB screening algorithm**<br>☐ Maximum age_____ (years)<br>☐ NA (no pediatric patients)

12.4 Did this HIV clinic diagnose TB disease in adult and/or pediatric HIV patients in 2019?	☐ Yes<br>☐ No **{→SKIP TO 12.7}**
12.5 For each of the following diagnostic tools, please indicate whether it was used in 2019 to evaluate **TB disease or infection** in adult patients, children, adults and children, or for none.	**TB diagnostics used to evaluate….**
a. AFB Smear	☐ Adults ☐ Children ☐ Adults & children ☐ None
b. Gene Xpert	☐ Adults ☐ Children ☐ Adults & children ☐ None
c. Chest X-ray	☐ Adults ☐ Children ☐ Adults & children ☐ None
d. Culture	☐ Adults ☐ Children ☐ Adults & children ☐ None
e. Urine LAM	☐ Adults ☐ Children ☐ Adults & children ☐ None
f. TB drug resistance testing	☐ Adults ☐ Children ☐ Adults & children ☐ None
g. Tuberculin skin testing (TST)/PPD for latent TB infection (LTBI)	☐ Adults ☐ Children ☐ Adults & children ☐ None
h. IGRA (e.g., Quantiferon Gold, T-spot) for latent TB infection (LTBI)	☐ Adults ☐ Children ☐ Adults & children ☐ None
i. Other (Specify)	☐ Adults ☐ Children ☐ Adults & children ☐ None

QUESTIONS	RESPONSES
**TB SCREENING, DIAGNOSIS AND PREVENTIVE THERAPY (CONTINUED)** *Describe practices/service delivery prior to COVID-19*	
12.6 For each of the following types of samples used for **microbiological diagnosis of TB**, please indicate whether they were collected in 2019 for adult patients, children, adults and children, or for none.	**Samples collected for ....**
a. Expectorated sputum	☐ Adults ☐ Children ☐ Adults & children ☐ None
b. Induced sputum	☐ Adults ☐ Children ☐ Adults & children ☐ None
c. Gastric aspirates	☐ Adults ☐ Children ☐ Adults & children ☐ None
d. Urine	☐ Adults ☐ Children ☐ Adults & children ☐ None
e. Biopsy	☐ Adults ☐ Children ☐ Adults & children ☐ None
f. "String test"	☐ Children ☐ None
g. Other (Specify)	☐ Adults ☐ Children ☐ Adults & children ☐ None

12.7 During 2019, where were the following **TB diagnostic tests** typically performed for adult and/or pediatric patients enrolled in care at this HIV clinic? *Confirm whether specimen collection/diagnostics were performed in the HIV clinic, elsewhere at the same health facility, only off-site or are not available for routine patient care. Select one best response.*	Provided in HIV Clinic	Elsewhere in health facility	Only offsite (referral)	Not available
a. AFB Smear	☐	☐	☐	☐
b. Gene Xpert	☐	☐	☐	☐
c. Chest X-ray	☐	☐	☐	☐
d. Culture	☐	☐	☐	☐
e. Urine LAM	☐	☐	☐	☐
f. TB drug resistance testing	☐	☐	☐	☐
g. Tuberculin skin testing (TST)/PPD for latent TB infection (LTBI)	☐	☐	☐	☐
h. IGRA (e.g., Quantiferon Gold, T-spot) for latent TB infection (LTBI)	☐	☐	☐	☐
i. Other (Specify)	☐	☐	☐	☐

12.8 **In 2019, d**id **HIV patients typically pay any fee** (other than insurance co-pays) for the following screening/diagnostics for TB? *Select one best response.*	***Please indicate if patients pay fees other than insurance co-pays. Select NA for services not available for routine care.***
a. AFB Smear	☐ Yes ☐ No ☐ Do not know ☐ NA
b. Gene Xpert	☐ Yes ☐ No ☐ Do not know ☐ NA
c. Chest X-ray	☐ Yes ☐ No ☐ Do not know ☐ NA
d. Culture	☐ Yes ☐ No ☐ Do not know ☐ NA
e. Urine LAM	☐ Yes ☐ No ☐ Do not know ☐ NA
f. TB drug resistance testing	☐ Yes ☐ No ☐ Do not know ☐ NA
g. Tuberculin skin testing (TST)/PPD for latent TB infection (LTBI)	☐ Yes ☐ No ☐ Do not know ☐ NA
h. IGRA (e.g., Quantiferon Gold, T-spot) for latent TB infection (LTBI)	☐ Yes ☐ No ☐ Do not know ☐ NA
12.9 Which types of HIV patients can be **treated for TB disease** at this health facility (either within the HIV clinic or in a co-located TB clinic)? *Check all that apply. If patients are referred elsewhere for TB treatment, select "None."*	☐ None (all patients referred offsite for TB treatment) **{→SKIP TO Q12.15}**<br>☐ Adult patients<br>☐ Pediatric patients
12.10 In 2019, what type of tracing was performed for the **household contacts of HIV patients diagnosed with active TB**? *Select one best response.*	☐ No tracing or systematic documentation of contacts of active TB cases<br>☐ Site staff performed contact tracing and maintain a TB contact register<br>☐ Site staff recorded information about contacts of TB cases, but no dedicated register<br>☐ Contact tracing performed, but not by HIV clinic staff (e.g, performed by health department or a separate public health team)
12.11 In 2019, did this site (either the HIV clinic or a co-located TB clinic) confirm whether **household contacts of active TB cases were screened for TB and provided tuberculosis preventive therapy** (TPT), if TB was ruled out? *Select one best response.*	☐ Yes, the site maintained this information in a TB contact register<br>☐ Yes, the site documented this information, but not in a dedicated register<br>☐ No, this was done by a separate public health team (e.g. health department)<br>☐ No systematic documentation done for contacts of active TB cases
12.12 Did this site (either the HIV clinic or a co-located TB clinic) confirm whether **household contacts of active TB cases completed tuberculosis preventive therapy** (TPT)? *Select one best response.*	☐ Yes, the site maintained this information a TB contact register<br>☐ Yes, the site documented this information, but not in a dedicated register<br>☐ No, this was done by a separate public health team (e.g. health department)<br>☐ No, systematic documentation done for contacts of active TB cases

QUESTIONS	RESPONSES
**TB SCREENING, DIAGNOSIS, AND PREVENTIVE THERAPY (CONTINUED)** *Describe practices/service delivery prior to COVID-19*	
12.13 What is done to **track patients with TB disease who miss appointments**?<br>*Check all that apply, OR select "Nothing /No follow-up""*	☐ Nothing/No follow-up with patients with TB disease who miss appointments<br>☐ Phone call to individual and/or family<br>☐ Send message via letter, email, SMS, or online patient portal<br>☐ Home visit by clinic staff<br>☐ Home visit by community outreach worker<br>☐ Outreach by peer supporter/mentor<br>☐ Other (specify) ___
12.14 How are patients defined as lost to follow-up from **TB treatment**?<br>*Select one best response.*	☐ Do not know<br>☐ Treatment interruption for more than 2 weeks<br>☐ Treatment interruption for more than 1 month<br>☐ Treatment interruption for more than 2 months<br>☐ Treatment interruption for more than 3 months<br>☐ Other, specify: ___
12.15 In 2019, did this HIV clinic (or a co-located TB clinic) provide **TB preventive therapy (TPT)** for patients who screened negative for TB disease?	☐ Yes<br>☐ No **{→SKIP TO Q12.22}**

12.16 Please indicate whether the following **TB preventive therapy (TPT) eligibility criteria** were used in 2019 with adult patients, children, or adults & children, or with none**.**	**TPT eligibility criteria used for ….**
a. Patients newly diagnosed with HIV	☐ Adults ☐ Children ☐ Adults & children ☐ None
b. Patients currently receiving ART	☐ Adults ☐ Children ☐ Adults & children ☐ None
c. Patients with history of contact with TB case	☐ Adults ☐ Children ☐ Adults & children ☐ None
d. Patients who have not previously received TPT	☐ Adults ☐ Children ☐ Adults & children ☐ None
e. Patients who have previously been treated for TB disease	☐ Adults ☐ Children ☐ Adults & children ☐ None
f. Among non-pregnant adults, TST or IGRA positive only	☐ Adults ☐ None
g. All pregnant women	☐ Adults ☐ None
h. Among pregnant women, TST or IGRA positive only	☐ Adults ☐ None
i. Children under 5 years	☐ Children ☐ None
j. Children ages 6-15 years	☐ Children ☐ None
k. Among children, TST or IGRA positive only	☐ Children ☐ None
l. Children who are household contacts, regardless of TST or IGRA status	☐ Children ☐ None
m. Other (Specify)___	☐ Adults ☐ Children ☐ Adults & children ☐ None

12.17 Please indicate whether the following **TB preventive therapy (TPT) regimens** were provided in 2019 to adult patients, children, adults & children, or to none?	**TPT regimens provided for …..**
a. 6-month isoniazid (**6H**)	☐ Adults ☐ Children ☐ Adults & children ☐ None
b. 9-month isoniazid (**9H**)	☐ Adults ☐ Children ☐ Adults & children ☐ None
c. 12-month isoniazid (**12H**)	☐ Adults ☐ Children ☐ Adults & children ☐ None
d. 36/Lifetime isoniazid (**36/Lifetime H**)	☐ Adults ☐ Children ☐ Adults & children ☐ None
e. 3-month rifampicin (**3R**)	☐ Adults ☐ Children ☐ Adults & children ☐ None
f. 4-month rifampicin (**4R**)	☐ Adults ☐ Children ☐ Adults & children ☐ None
g. 3-month isoniazid-rifampicin (**3HR**)	☐ Adults ☐ Children ☐ Adults & children ☐ None
h. 4-month isoniazid-rifampicin (**4HR**)	☐ Adults ☐ Children ☐ Adults & children ☐ None
i. Once-weekly isoniazid-rifapentine for 12 weeks (**3HP**)	☐ Adults ☐ Children ☐ Adults & children ☐ None
j. Once-daily isoniazid-rifapentine for 1 month (**1HP**)	☐ Adults ☐ Children ☐ Adults & children ☐ None
k. Regimens for MDR-TB exposure (Specify)___	☐ Adults ☐ Children ☐ Adults & children ☐ None
l. Other (Specify)___	☐ Adults ☐ Children ☐ Adults & children ☐ None

12.18 Which contraindications are patients screened for prior to TPT initiation?<br>*Check all that apply, OR select "Not applicable"*	☐ Not applicable (patients are not screened for TPT contraindications)<br>☐ Jaundice, liver disease<br>☐ Numbness, tingling (peripheral neuropathy)<br>☐ Previous adverse reaction (e.g. hypersensitivity/flu-like symptoms, rash)<br>☐ Alcohol misuse<br>☐ Age<br>☐ TB disease<br>☐ Other (Specify) ___
12.19 Are HIV patients receiving TPT eligible for differentiated service delivery of HIV care?	☐ Yes<br>☐ No

QUESTIONS	RESPONSES
**TB SCREENING, DIAGNOSIS, AND PREVENTIVE THERAPY (CONTINUED)**	
12.20 Which signs/symptoms of adverse events are monitored in patients receiving TPT?<br>*Check all that apply, OR select "Not applicable."*	☐ Not applicable (patients receiving TPT are not monitored for adverse events<br>☐ Hepatitis symptoms (nausea, vomiting, abdominal pain)<br>☐ Numbness, tingling (peripheral neuropathy)<br>☐ Elevated liver enzymes<br>☐ Flu-like symptoms<br>☐ Rash<br>☐ Other (Specify) ____________________________
12.21 In 2019 what was done to track TPT patients with HIV who missed appointments?<br>*Check all that apply, OR select "Nothing/No follow-up."*	☐ Nothing/No follow-up with TPT patients who miss appointments<br>☐ Phone call to individual and/or family<br>☐ Send message via letter, email, SMS, or online patient portal<br>☐ Home visit by clinic staff<br>☐ Home visit by community outreach worker<br>☐ Outreach by peer supporter/mentor<br>☐ Other (specify) ______________________________________
12.22 Is there someone at this site who can be contacted for additional information about treatment of TB at this at this health facility?	☐ No **{→ SKIP TO 13.1}**<br>☐ Yes (please provide name and email)<br>Name: ____________________________<br>Email: ____________________________
**13. PATIENT SCREENING AND TREATMENT FOR SUBSTANCE USE DISORDERS**. *Describe practices prior to COVID-19*	
13.1 Are any HIV patients screened for **alcohol use disorders**?<br>*Screening refers to any type of structured or unstructured assessment.*	☐ Yes<br>☐ No **{→SKIP TO 13.6}**
13.2 Which HIV patients are screened for **alcohol use disorders**?<br>*Check all that apply.*	☐ All patients<br>☐ Patients with symptoms of possible alcohol use disorders<br>☐ Patients with therapeutic failure<br>☐ Patients who are not adherent to ART<br>☐ Other types of patients (specify)__________________
13.3 Which structured instrument(s) are used to screen patients for **alcohol use disorders**?<br>*Check all that apply.*	☐ None (no structured or standardized screening tool used)<br>☐ Alcohol Use Disorders Identification Test (AUDIT)<br>☐ Alcohol Use Disorders Identification Test-C (AUDIT-C)<br>☐ Alcohol, Smoking, and Substance Involvement Screening Test (ASSIST)<br>☐ Cut down, Annoyed, Guilty, Eye-opener (CAGE)<br>☐ Other (specify)_____________________
13.4 Which of the following biomarkers can be assessed at this health facility in screening for alcohol use disorders?<br>*Check all that apply.*	☐ None – biomarkers not used in screening for alcohol use disorders<br>☐ Aspartate transaminase (AST)<br>☐ Aspartate transaminase, Alanine transaminase ratio (AST/ALT)<br>☐ Blood alcohol concentration (BAC)<br>☐ Ethyl glucuronide (EtG)<br>☐ Other (specify)_____________
13.5 For patients who screen positive for **alcohol use disorders**, what treatment interventions are available at this health facility?<br>*Check all that apply.*	☐ None (no treatment available at this health facility)<br>☐ Counseling<br>☐ Brief Intervention<br>☐ Detox hospitalization<br>☐ Pharmacological treatment (Disulfiram, Naltrexone, Acamprosate)<br>☐ Psychotherapy (motivational interview, cognitive-behavioral therapy (CBT), relapse prevention)<br>☐ Screening, Brief Intervention, and Referral to Treatment (SBIRT)<br>☐ Other (specify) ________________
13.6 Which of the following **other substance use disorders** are patients screened for in the HIV clinic?<br>*Screening refers to any type of structured or unstructured assessment.*<br>*Check all that apply.*	☐ None **{→SKIP TO 14.1}**<br>☐ Cannabis (marijuana)<br>☐ Cocaine/crack<br>☐ Ecstasy and other club drugs<br>☐ Hallucinogens<br>☐ Methamphetamine<br>☐ Opioids<br>☐ Other (specify): ____

QUESTIONS	RESPONSES
**PATIENT SCREENING AND TREATMENT FOR SUBSTANCE USE DISORDERS (CONTINUED)** *Describe practices prior to COVID-19*	
13.7 Which patients are screened for **other substance use disorders**?<br>*Check all that apply.*	☐ All patients<br>☐ Patients with symptoms of possible drug use disorders<br>☐ Patients with therapeutic failure<br>☐ Patients who are not adherent to ART<br>☐ Other types of patients (specify)__________________
13.8 Which structured instrument(s) are used to screen patients for **substance use disorders** (other than alcohol use)?<br>*Check all that apply.*	☐ None (no structured or standardized screening tool used)<br>☐ Addiction Severity Index (ASI)<br>☐ Alcohol, Smoking, and Substance Involvement Screening Test (ASSIST)<br>☐ Drug Abuse Screening Test (DAST)<br>☐ Other (specify)____________________
13.9 For patients who screen positive for **substance use disorders** (other than alcohol use) what treatment interventions are available at this health facility?<br>*Check all that apply.*	☐ None (no treatment available at this health facility)<br>☐ Counseling<br>☐ Brief Intervention<br>☐ Detox hospitalization<br>☐ Methadone replacement therapy<br>☐ Pharmacological treatment<br>☐ Psychotherapy (motivational interview, cognitive-based therapy (CBT), relapse prevention)<br>☐ Screening, Brief Intervention, and Referral to Treatment (SBIRT)<br>☐ Syringe exchange<br>☐ Other (specify) ________________
**14. PATIENT SCREENING AND TREATMENT FOR MENTAL HEALTH DISORDERS.** *Describe practices prior to COVID-19*	
14.1 Are any HIV patients screened for **depression**?<br>*Screening refers to any type of structured or unstructured assessment.*	☐ Yes<br>☐ No **{→SKIP TO 14.5}**
14.2 Which patients are screened for **depression**?<br>*Check all that apply.*	☐ All patients, including those not presenting with mental health symptoms<br>☐ Patients presenting with mental health symptoms<br>☐ Patients with therapeutic failure<br>☐ Patients who are not adherent to ART<br>☐ Other types of patients (specify)__________________
14.3 Which structured instrument(s) are used to screen patients for **depression**?<br>*Check all that apply.*	☐ None (no structured or standardized depression screening tool used)<br>☐ Beck Depression Inventory (BDI)<br>☐ Center for Epidemiologic Studies Depression Scale (CES-D)<br>☐ Hamilton Rating Scale for Depression (HAM-D)<br>☐ Hospital Anxiety and Depression Scale (HAD)<br>☐ Patient Health Questionnaire-2 (PHQ-2)<br>☐ Patient Health Questionnaire-9 (PHQ-9)<br>☐ Other (specify)____________________
14.4 For patients who screen positive for depression, what treatment interventions are available at this health facility?<br>*Check all that apply.*	☐ None (no treatment available at this health facility)<br>☐ Individual counseling or psychotherapy<br>☐ Group counseling or psychotherapy<br>☐ Medication<br>☐ Peer support<br>☐ Psychosocial support<br>☐ Other (specify)_______________
14.5 Are any HIV patients screened for **post-traumatic stress disorder (PTSD)**?	☐ Yes<br>☐ No **{→SKIP TO 14.9}**
14.6 Which patients are screened for **PTSD**?<br>*Check all that apply.*	☐ All patients, including those not presenting with mental health symptoms<br>☐ Patients presenting with mental health symptoms<br>☐ Patients with therapeutic failure<br>☐ Patients who are not adherent to ART<br>☐ Other types of patients (specify)__________________
14.7 Which structured instrument(s) are used to screen patients for **PTSD**?<br>*Check all that apply.*	☐ None (no structured or standardized PTSD screening tool used)<br>☐ Life Event Checklist<br>☐ Primary Care PTSD Screen (PC-PTSD)<br>☐ PTSD Checklist – Civilian version (PCL-C)<br>☐ PTSD Checklist for DSM-5 (PCL-5)<br>☐ Short PTSD Rating Interview (SPRINT)<br>☐ Trauma Screening Questionnaire (TSQ)<br>☐ Other (specify)____________________

QUESTIONS	RESPONSES
**PATIENT SCREENING AND TREATMENT FOR MENTAL HEALTH DISORDERS (CONTINUED)** *Describe practices prior to COVID-19*	
14.8 For patients who screen positive for **PTSD**, what treatment interventions are available at this health facility? *Check all that apply.*	☐ None (no treatment available at this health facility)<br>☐ Individual counseling or psychotherapy<br>☐ Group counseling or psychotherapy<br>☐ Medication<br>☐ Peer support<br>☐ Psychosocial support<br>☐ Other (specify) ________________
14.9 Are any HIV patients screened for **anxiety disorders** (other than PTSD)?	☐ Yes<br>☐ No **{→SKIP TO 14.13}**
14.10 Which patients are screened for **anxiety disorders**? *Check all that apply.*	☐ All patients, including those not presenting with mental health symptoms<br>☐ Patients presenting with mental health symptoms<br>☐ Patients with therapeutic failure<br>☐ Patients who are not adherent to ART<br>☐ Other types of patients (specify)__________________
14.11 Which structured instrument(s) are used to screen patients for **anxiety disorders**? *Check all that apply.*	☐ None (no structured or standardized screening tool used)<br>☐ Beck Anxiety Inventory (BAI)<br>☐ Generalized Anxiety Disorder 7-item scale (GAD-7)<br>☐ Hospital Anxiety and Depression Scale (HAD)<br>☐ State-Trait Anxiety Inventory (STAI)<br>☐ Other (specify)_____________________
14.12 For patients who screen positive for **anxiety disorders** (other than PTSD), what treatment interventions are available at this health facility? *Check all that apply.*	☐ None (no treatment available at this health facility)<br>☐ Individual counseling or psychotherapy<br>☐ Group counseling or psychotherapy<br>☐ Medication<br>☐ Peer support<br>☐ Psychosocial support<br>☐ Other (specify)________________
14.13 Are HIV patients screened for any of the following mental health conditions? *Check all that apply.*	☐ Other mental health disorders (bipolar, schizophrenia, etc.)<br>☐ Cognitive impairment<br>☐ Suicide risk<br>☐ None of the above
14.14 Is there a standard safety protocol for responding to patients with suicidal or homicidal intentions?	☐ Yes<br>☐ No

**15. DIAGNOSIS OF KAPOSI'S SARCOMA.** *Describe practices/service delivery prior to COVID-19.*				
15.1 During 2019, were any patients diagnosed with Kaposi's sarcoma at this site (either the HIV clinic or another unit of the health facility)?	☐ Yes<br>☐ No **{→SKIP TO 16.1}**			
15.2 During 2019, which of the following procedures were used to diagnose Kaposi's sarcoma and where was the procedure performed for HIV patients? *Check all that apply.*	**In HIV Clinic**	**Outpatient or inpatient ward**	**Operating theatre**	**Not available**
a. Clinical exam (visual inspection of skin or mucosal surfaces)	☐	☐	☐	☐
b. Skin punch biopsy	☐	☐	☐	☐
c. Surgical wedge/excision	☐	☐	☐	☐
d. Other (specify):	☐	☐	☐	☐

15.3 In 2019, did HIV patients **typically pay any fee (other than insurance co-pays)** for the following procedures for diagnosing Kaposi's sarcoma?	***Please indicate if patients paid fees other than insurance co-pays. Select NA for services not available for routine care.***
a. Clinical exam (visual inspection of skin or mucosal surfaces)	☐ Yes ☐ No ☐ Do not know ☐ NA
b. Skin punch biopsy	☐ Yes ☐ No ☐ Do not know ☐ NA
c. Surgical wedge/excision	☐ Yes ☐ No ☐ Do not know ☐ NA
d. Other (specify):	☐ Yes ☐ No ☐ Do not know ☐ NA
15.4. Is there someone who can be contacted for additional information about diagnosis and treatment of Kaposi's sarcoma at this health facility?	☐ No<br>☐ Yes (please provide name and email)<br>Name:<br>Email:

QUESTIONS	RESPONSES	
**16. PHARMACY.** *Describe service delivery prior to COVID-19.*		
16.1. Is there a pharmacy located at this health facility?	☐ Yes<br>☐ No **{→ SKIP TO 17.1}**	
16.2 For each of the following medications, please indicate whether they were **dispensed/available at this health facility** during 2019 and whether there were **supply disruptions/stock-outs lasting at least one week during 2019**	**Medication dispensed in 2019**	**Stock-out lasting at least 1 week in 2019.** Select NA if not dispensed
a. First-line HIV antiretroviral medications (ARVs)	☐ Yes ☐ No	☐ Yes ☐ No ☐ NA
b. Second-line HIV ARVs	☐ Yes ☐ No	☐ Yes ☐ No ☐ NA
c. Third-line HIV ARVs	☐ Yes ☐ No	☐ Yes ☐ No ☐ NA
d. Isoniazid	☐ Yes ☐ No	☐ Yes ☐ No ☐ NA
e. Rifapentine	☐ Yes ☐ No	☐ Yes ☐ No ☐ NA
f. TB medications other than isoniazid and rifapentine	☐ Yes ☐ No	☐ Yes ☐ No ☐ NA
g. Cotrimoxazole (Bactrim, Septra, TMP-SMX)	☐ Yes ☐ No	☐ Yes ☐ No ☐ NA
h. Malaria treatment	☐ Yes ☐ No	☐ Yes ☐ No ☐ NA
i. Fluconazole	☐ Yes ☐ No	☐ Yes ☐ No ☐ NA
j. Amphotericin B	☐ Yes ☐ No	☐ Yes ☐ No ☐ NA
k. Flucytosine (5FC)	☐ Yes ☐ No	☐ Yes ☐ No ☐ NA
l. Short-acting contraceptives (pills, injectables, condoms)	☐ Yes ☐ No	☐ Yes ☐ No ☐ NA
m. Long-acting reversible contraceptives (implants, intrauterine devices)	☐ Yes ☐ No	☐ Yes ☐ No ☐ NA
n. Selective serotonin reuptake inhibitors (SSRIs: e.g., Prozac, Zoloft, Paxil)	☐ Yes ☐ No	☐ Yes ☐ No ☐ NA
o. Serotonin and norepinephrine reuptake inhibitors (SNRIs: e.g., Cymbalta, Effexor, Fetzima)	☐ Yes ☐ No	☐ Yes ☐ No ☐ NA
p. Tricyclic Antidepressants (e.g., amitriptyline, amoxapine, doxepin)	☐ Yes ☐ No	☐ Yes ☐ No ☐ NA
q. Benzodiazepines (e.g., Xanax, Lorazepam, Klonopin)	☐ Yes ☐ No	☐ Yes ☐ No ☐ NA
r. Antipsychotic medications (e.g., Haloperidol, Chlorpromazine, Fluphenazine, Risperidone, Seroquel, Abilify)	☐ Yes ☐ No	☐ Yes ☐ No ☐ NA
s. Mood stabilizers (e.g., Carbamazepine, Lithium, Valproate, Lamotrigine)	☐ Yes ☐ No	☐ Yes ☐ No ☐ NA
t. Alcohol dependence medications (Disulfiram, Naltrexone, Acamprosate)	☐ Yes ☐ No	☐ Yes ☐ No ☐ NA
16.3 In 2019, did this HIV clinic have patients on a **waiting list to receive ART**?	☐ Yes<br>☐ No<br>☐ Don't know	
**17. MEDICAL RECORDS AND PATIENT TRACKING.** *Describe practices prior to COVID-19.*		
17.1. In 2019, did this clinic track the outcomes of HIV patients who were lost to follow-up (e.g. outcomes such as patient deaths, transfers to other facilities, ART status, etc.)?	☐ Yes<br>☐ No **{→ SKIP TO 18.1}**	
17.2 Were the outcomes of tracked patients recorded in electronic databases?	☐ Yes<br>☐ No **{→ SKIP TO 18.1}**	
17.3. After tracking patients lost to follow-up at this HIV clinic, what information was recorded in electronic databases?<br>*Check all that apply.*	☐ Transfers to other facilities<br>☐ Loss to follow-up<br>☐ Deaths<br>☐ Other (specify) ___________________	

**18. COVID-19 RESPONSE & IMPACT ON HIV CARE AND TREATMENT.**<br>*Describe how COVID-19 has affected HIV service delivery at this clinic.*	
18.1 Was the geographic location surrounding this HIV clinic subject to any form of COVID-19 restrictions on travel, service provision, or business operations?	☐ Yes<br>☐ No **{SKIP TO Q18.2}**
18.1a When were COVID-19-related restrictions first issued for the geographic location surrounding this HIV clinic? *Please provide month. If unknown, select DO NOT KNOW.*	☐ MM/ 2020<br>☐ Do not know
18.1b When were COVID-19-related restrictions first lifted or eased?<br>*Please provide month. If unknown, select DO NOT KNOW.*<br>*If restrictions remain in place, record NA - not applicable.*	☐ MM/ 2020<br>☐ Do not know<br>☐ NA (not applicable)
18.2 Did this HIV clinic suspend the provision of HIV services in response to COVID-19?	☐ Yes<br>☐ No **{SKIP TO Q18.3}**
18.2a When were HIV-related services first suspended at this hospital/clinic?<br>*Please provide month. If unknown, select DO NOT KNOW.*	☐ MM/ 2020<br>☐ Do not know
18.2b When were HIV-related services first resumed at this hospital/clinic?<br>*Please provide month. If unknown, select DO NOT KNOW.*<br>*If HIV-related services remain suspended, record NA - not applicable.*	☐ MM/ 2020<br>☐ Do not know<br>☐ NA (not applicable)

QUESTIONS	RESPONSES
**COVID-19 RESPONSE & IMPACT** (continued)	
18.3 At any time since the start of the pandemic, has the COVID-19 response resulted in any of the following changes in the **operations of the HIV clinic**, and are any of these changes currently in effect?<br>*Please indicate whether the following changes are **currently, previously**, or **never** in effect at this HIV clinic. Select NA (not applicable) for operations (e.g. HIV testing, research, etc.) that were not in place prior to the COVID-19 pandemic.*	
a. Suspension or postponement of the enrollment of new patients in HIV care	☐ Currently ☐ Previously ☐ Never
b. Suspension or postponement of non-urgent appointments for HIV patients	☐ Currently ☐ Previously ☐ Never
c. Decreases in the number of hours or days of service delivery for HIV patients	☐ Currently ☐ Previously ☐ Never
d. Re-assignment of HIV care providers to assist with the COVID-19 response	☐ Currently ☐ Previously ☐ Never
e. Reduced availability of HIV care providers due to COVID-19-related illness, self-isolation, or quarantine	☐ Currently ☐ Previously ☐ Never
f. Reconfiguration of hospital/clinic space to accommodate COVID-19-related services	☐ Currently ☐ Previously ☐ Never
g. Increased use of personal protective equipment (masks, gloves, gowns, etc.) by HIV clinic staff	☐ Currently ☐ Previously ☐ Never
h. Increased use of telemedicine (i.e., consultations by phone/web) in HIV-related care	☐ Currently ☐ Previously ☐ Never
i. Interruptions or changes in recording of data (either paper or electronic records) related to clinical management of patients	☐ Currently ☐ Previously ☐ Never
j. Suspension or decreases in the availability of HIV testing/diagnostic services	☐ Currently ☐ Previously ☐ Never ☐ NA
k. Suspension or postponement of ongoing research activities (e.g., enrollment or follow-up of patients in ongoing research studies)	☐ Currently ☐ Previously ☐ Never ☐ NA
l. Interruptions or changes in recording of data (either paper or electronic records) for ongoing research	☐ Currently ☐ Previously ☐ Never ☐ NA
m. Interruptions or delays in initiation of or planning for new research activities unrelated to COVID-19	☐ Currently ☐ Previously ☐ Never ☐ NA
n. Withdrawal/suspension of activities of non-governmental partners that support care provision in the clinic	☐ Currently ☐ Previously ☐ Never ☐ NA
18.4 At any time since the start of the pandemic, has the COVID-19 response resulted in partial or complete suspension of any of the following **community-based HIV services** (i.e., services provided in community settings outside the hospital/clinic) for patients referred to or enrolled in care at this HIV clinic?<br>*Please indicate whether the following community-based services are **currently**, **previously** or **never** suspended. Select NA (not applicable) for community activities that were not in place prior to the COVID-19 pandemic.*	
a. Community-based HIV testing	☐ Currently ☐ Previously ☐ Never ☐ NA
b. Community-based ART refills	☐ Currently ☐ Previously ☐ Never ☐ NA
c. Community-based support group meetings/activities	☐ Currently ☐ Previously ☐ Never ☐ NA
d. Community-based tracing of patients who are lost to follow-up (LTFU)	☐ Currently ☐ Previously ☐ Never ☐ NA
e. Withdrawal/suspension of activities of non-governmental partners that support community-based programs for patients enrolled in HIV care at this clinic	☐ Currently ☐ Previously ☐ Never ☐ NA
18.5 At any time since the start of the pandemic, have **routine ART services** at this HIV clinic been impacted by COVID-19?<br>*Please indicate whether the following changes are **currently, previously**, or **never** experienced at this HIV clinic. Select NA (not applicable) for services that were not in place prior to the COVID-19 pandemic.*	
a. ART clinics have been suspended or shut down	☐ Currently ☐ Previously ☐ Never ☐ NA
b. ART pick-up points have been designated in the community	☐ Currently ☐ Previously ☐ Never ☐ NA
c. Patients are being given extra supplies/refills of ART to reduce the frequency of refills.	☐ Currently ☐ Previously ☐ Never ☐ NA
d. Other (specify)________________________	☐ Currently ☐ Previously ☐ Never ☐ NA
18.6 At any time since the start of the pandemic, have **ART initiation services** at this HIV clinic been impacted by COVID-19?<br>*Please indicate whether the following impacts are **currently, previously**, or **never** experienced at this HIV clinic. Select NA (not applicable) for services that were not available prior to the COVID-19 pandemic.*	
a. ART initiation services have been suspended	☐ Currently ☐ Previously ☐ Never ☐ NA
b. Same-day or rapid ART initiation services introduced or expanded	☐ Currently ☐ Previously ☐ Never ☐ NA
c. Adherence counseling requirements prior to ART initiation reduced or streamlined.	☐ Currently ☐ Previously ☐ Never ☐ NA
d. Other (specify)________________________	☐ Currently ☐ Previously ☐ Never ☐ NA

QUESTIONS	RESPONSES
**COVID-19 RESPONSE & IMPACT** (continued)	
18.7 At any time since the start of the pandemic, have **HIV viral load testing services** at this HIV clinic been impacted by COVID-19? *Please indicate whether the following impacts are **currently, previously**, or **never** experienced at this HIV clinic. Select NA (not applicable) for services that were not available prior to the COVID-19 pandemic.*	
a. Sample collection for HIV viral load testing has been suspended	☐ Currently ☐ Previously ☐ Never ☐ NA
b. Laboratory not accepting HIV viral load samples	☐ Currently ☐ Previously ☐ Never ☐ NA
c. Turnaround time for HIV viral load testing is longer	☐ Currently ☐ Previously ☐ Never ☐ NA
d. Other (specify)____	☐ Currently ☐ Previously ☐ Never ☐ NA
18.8 At any time since the start of the pandemic, have, has the HIV clinic experienced stockouts of any of the following for care of HIV patients? *Please indicate whether the following stockouts are **currently** and/or **previously** experienced, or **never** experienced. Select NA (not applicable) for commodities and supplies that were not routinely available/provided at this clinic prior to the start of the COVID-19 pandemic.*	
a. HIV test kits	☐ Currently ☐ Previously ☐ Never ☐ NA
b. PrEP medications	☐ Currently ☐ Previously ☐ Never ☐ NA
c. First-line antiretroviral regimens	☐ Currently ☐ Previously ☐ Never ☐ NA
d. Second-line antiretroviral regimens	☐ Currently ☐ Previously ☐ Never ☐ NA
e. Third-line antiretroviral regimens	☐ Currently ☐ Previously ☐ Never ☐ NA
f. Supplies for viral load testing	☐ Currently ☐ Previously ☐ Never ☐ NA
**19. ACKNOWLEDGEMENTS (OPTIONAL)**	
19.1. We would like to acknowledge clinic team members who participated in the completion of this survey. If your team members would like their names included, please enter their full names, separated by commas, so we can acknowledge their contribution.	

9-SEP-20

Thank you for your participation.
