# Supplementary material for: Design and implementation of a global site assessment survey among HIV clinics participating in the International epidemiology Databases to Evaluate AIDS (IeDEA) research consortium
Source: PLoS One. 2023 Mar 14;18(3):e0268167. doi: 10.1371/journal.pone.0268167 (PMC10013879; doi:10.1371/journal.pone.0268167)
Supplement: S3 Text — (DOCX) [file pone.0268167.s003.docx]

# S3 Text. IeDEA Study membership

**IeDEA Asia-Pacific**

## Site investigators and study teams:

**The TREAT Asia HIV Observational Database:** V Khol, V Ouk, C Pov, National Center for HIV/AIDS, Dermatology & STDs, Phnom Penh, Cambodia; FJ Zhang, HX Zhao, N Han, Beijing Ditan Hospital, Capital Medical University, Beijing, China; MP Lee, PCK Li, TS Kwong, TH Li, Queen Elizabeth Hospital, Hong Kong SAR, China; N Kumarasamy, C Ezhilarasi, Chennai Antiviral Research and Treatment Clinical Research Site (CART CRS), VHS-Infectious Diseases Medical Centre, VHS, Chennai, India; S Pujari, K Joshi, S Gaikwad, A Chitalikar, Institute of Infectious Diseases, Pune, India; IKA Somia, TP Merati, AAS Sawitri, F Yuliana, Faculty of Medicine Udayana University & Sanglah Hospital, Bali, Indonesia; E Yunihastuti, A Widhani, S Maria, TH Karjadi, Faculty of Medicine Universitas Indonesia - Dr. Cipto Mangunkusumo General Hospital, Jakarta, Indonesia; J Tanuma, S Oka, T Nishijima, National Center for Global Health and Medicine, Tokyo, Japan; JY Choi, Na S, JM Kim, Division of Infectious Diseases, Department of Internal Medicine, Yonsei University College of Medicine, Seoul, South Korea; YM Gani, NB Rudi, Hospital Sungai Buloh, Sungai Buloh, Malaysia; I Azwa, A Kamarulzaman, SF Syed Omar, S Ponnampalavanar, University Malaya Medical Centre, Kuala Lumpur, Malaysia; R Ditangco, MK Pasayan, ML Mationg, Research Institute for Tropical Medicine, Muntinlupa City, Philippines; YJ Chan, HP Chen, PF Wu, E Ke, Taipei Veterans General Hospital, Taipei, Taiwan; OT Ng, PL Lim, LS Lee, T Yap, Tan Tock Seng Hospital, Singapore; A Avihingsanon, S Gatechompol, P Phanuphak, C Phadungphon, HIV-NAT/Thai Red Cross AIDS Research Centre, Bangkok, Thailand; S Kiertiburanakul, A Phuphuakrat, L Chumla, N Sanmeema, Faculty of Medicine Ramathibodi Hospital, Mahidol University, Bangkok, Thailand; R Chaiwarith, T Sirisanthana, J Praparattanapan, K Nuket, Chiang Mai University - Research Institute for Health Sciences, Chiang Mai, Thailand; S Khuwuwan, P Payoong, P Kantipong, P Kambua, Chiangrai Prachanukroh Hospital, Chiang Rai, Thailand; TN Pham, KV Nguyen, DTH Nguyen, DT Nguyen, National Hospital for Tropical Diseases, Hanoi, Vietnam; CD Do, AV Ngo, LT Nguyen, Bach Mai Hospital, Hanoi, Vietnam; AH Sohn, JL Ross, B Petersen, TREAT Asia, amfAR - The Foundation for AIDS Research, Bangkok, Thailand; MG Law, A Jiamsakul, D Rupasinghe, The Kirby Institute, UNSW Sydney, NSW, Australia.

**The Australian HIV Observational Database:** *New South Wales:* M Bloch, T Vincent, Holdsworth House Medical Practice, Sydney; D Smith, N Edmiston, L Lindsay,, Lismore Sexual Health & AIDS Services, Lismore; D Baker, R Mousavi, S Cabot, East Sydney Doctors, Surry Hills; DJ Templeton, L Garton, T Doyle, RPA Sexual Health, Camperdown; N Ryder, G Sweeney, B Moran, Clinic 468, HNE Sexual Health, Tamworth; A Carr, K Hesse, J Rogers, St Vincent’s Hospital, Darlinghurst; R Finlayson, L Tan, A Ahmad; M Alfonso, R Cossetto, Taylor Square Private Clinic, Darlinghurst; R Bopage, J Walsh, Western Sydney Sexual Health Clinic; A Cogle, National Association of People living with HIV/AIDS; C Lawrence, National Aboriginal Community Controlled Health Organisation; M Law, K Petoumenos, N Rose, J Hutchinson, S Virachit, T Dougherty, The Kirby Institute, University of NSW. *Northern Territory:* M Gunathilake, K Carlton, Centre for Disease Control, Darwin*. Queensland:* C Thng, Gold Coast Sexual Health Clinic, Southport; D Russell, F Bassett, M Rodriguez, Cairns Sexual Health Service, Cairns; D Sowden, K Taing, P Smith, Clinic 87, Sunshine Coast Hospital and Health Service, Nambour; J Langton-Lockton, E Knowland, Sexual Health and HIV Service in Metro North, Brisbane; *Victoria:* R Moore, Northside Clinic, North Fitzroy; NJ Roth, H Lau, Prahran Market Clinic, South Yarra; R Teague, J Silvers, W Zeng, Melbourne Sexual Health Centre, Melbourne; J Hoy, M Giles, M Bryant, S Price, P Rawson-Harris, The Alfred Hospital, Melbourne; I Woolley, T Korman, J O’Bryan, K Cisera, Monash Medical Centre, Clayton.

**The TREAT Asia Pediatric HIV Observational Database:** V Khol, O Vichea, C Pov, National Centre for HIV/AIDS, Dermatology and STDs, Phnom Penh, Cambodia; J Tucker, New Hope for Cambodian Children, Phnom Penh, Cambodia; N Kumarasamy, E Chandrasekaran, Chennai Antiviral Research and Treatment Clinical Research Site (CART CRS), VHS-Infectious Diseases Medical Centre, VHS, Chennai, India; A Kinikar, V Mave, S Nimkar, I Marbaniang, BJ Medical College and Sassoon General Hospitals, Maharashtra, India; DK Wati, D Vedaswari, IB Ramajaya, Sanglah Hospital, Udayana University, Bali, Indonesia; N Kurniati, D Muktiarti, Cipto Mangunkusumo – Faculty of Medicine Universitas Indonesia, Jakarta, Indonesia; SM Fong, M Lim, F Daut, Hospital Likas, Kota Kinabalu, Malaysia; NK Nik Yusoff, P Mohamad, Hospital Raja Perempuan Zainab II, Kelantan, Malaysia; TJ Mohamed, MR Drawis, Department of Pediatrics, Women and Children Hospital Kuala Lumpur, Kuala Lumpur, Malaysia; R Nallusamy, KC Chan, Penang Hospital, Penang, Malaysia; T Sudjaritruk, V Sirisanthana, L Aurpibul, Department of Pediatrics, Faculty of Medicine, and Research Institute for Health Sciences, Chiang Mai University, Chiang Mai, Thailand; P Ounchanum, R Hansudewechakul, S Denjanta, A Kongphonoi, Chiangrai Prachanukroh Hospital, Chiang Rai, Thailand; P Lumbiganon, P Kosalaraksa, P Tharnprisan, T Udomphanit, Division of Infectious Diseases, Department of Pediatrics, Faculty of Medicine, Khon Kaen University, Khon Kaen, Thailand; G Jourdain, PHPT-IRD UMI 174 (Institut de recherche pour le développement and Chiang Mai University), Chiang Mai, Thailand; T Puthanakit, S Anugulruengkit, W Jantarabenjakul, R Nadsasarn, Department of Pediatrics and Center of Excellence for Pediatric Infectious Diseases and Vaccines, Faculty of Medicine, Chulalongkorn University, Bangkok, Thailand; K Chokephaibulkit, K Lapphra, W Phongsamart, S Sricharoenchai, Department of Pediatrics, Faculty of Medicine Siriraj Hospital, Mahidol University, Bangkok, Thailand; QT Du, KH Truong, CH Nguyen, Children’s Hospital 1, Ho Chi Minh City, Vietnam; VC Do, TM Ha, VT An Children’s Hospital 2, Ho Chi Minh City, Vietnam; LV Nguyen, DM Tran, HTT Tran, TTT Giang, National Hospital of Pediatrics, Hanoi, Vietnam; ON Le, Worldwide Orphans Foundation, Ho Chi Minh City, Vietnam; AH Sohn, JL Ross, T Suwanlerk, TREAT Asia/amfAR - The Foundation for AIDS Research, Bangkok, Thailand; MG Law, A Kariminia, The Kirby Institute, UNSW Sydney, NSW, Australia.

# IeDEA Caribbean, Central, and South America (CCASAnet):

**Fundación Huésped, Argentina:** Pedro Cahn, Carina Cesar, Valeria Fink, Zulma Ortiz, Florencia Cahn, Agustina Roldan, Ines Aristegui, Claudia Frola.

**Instituto Nacional de Infectologia-Fiocruz, Brazil:** Beatriz Grinsztejn, Valdilea G. Veloso, Paula M. Luz, Sandra Cardoso Wagner, Ruth Friedman, Ronaldo I. Moreira, Lara Esteves Coelho, Monica Derrico Pedrosa, Guilherme Amaral Calvet, Hugo Perazzo, Rodrigo Moreira, Maria Pia Diniz Ribeiro, Mario Sergio Pereira, Emilia Moreira Jalil.

**Universidade Federal de Minas Gerais, Brazil:** Jorge Pinto, Flavia Ferreira, Marcelle Maia.

**Universidade Federal de São Paulo, Brazil:** Regina Célia de Menezes Succi, Daisy Maria Machado, Aida de Fátima Barbosa Gouvêa, Fabiana do Carmo.

**Fundación Arriarán, Chile**: Claudia Cortes, Marcelo Wolff, Maria Fernanda Rodriguez, Gabriel Castillo, Gladys Allendes.

**Les Centres GHESKIO, Haiti:** Jean William Pape, Vanessa Rouzier, Adias Marcelin, Youry Macius, Stephano Saint Preux.

**Hospital Escuela Universitario, Honduras**: Marco Tulio Luque, Diana Varela, Magda Chavez, Ada Mailhot.

**Instituto Hondureño de Seguridad Social, Honduras:** Denis Padgett.

**Instituto Nacional de Ciencias Médicas y Nutrición Salvador Zubirán, Mexico**: Juan Sierra Madero, Brenda Crabtree Ramirez, Yanink Caro Vega.

**Instituto de Medicina Tropical Alexander von Humboldt, Peru**: Eduardo Gotuzzo, Fernando Mejia, Gabriela Carriquiry.

**Vanderbilt University Medical Center, USA:** Catherine C McGowan, Stephany N Duda, Bryan E Shepherd, Timothy Sterling, Anna K Person, Peter F Rebeiro, Jessica Castilho, William C Wester, Kate Clouse, Karu Jayathilake, Fernanda Maruri, Hilary Vansell, Marina Cruvinel Figueiredo, Cathy Jenkins, Ahra Kim, Sarah Lotspeich, Paridhi Ranadive.

**Vanderbilt University, USA:** Kate Clouse

# Central Africa (CA-IeDEA)

## CA-IeDEA Site investigators and cohorts:

Nimbona Pélagie, Association Nationale de Soutien aux Séropositifs et Malade du Sida (ANSS), Burundi; Patrick Gateretse, Jeanine Munezero, Valentin Nitereka, Annabelle Niyongabo, Zacharie Ndizeye , Christella Twizere, Théodore Niyongabo, Centre National de Référence en Matière de VIH/SIDA, Burundi; Hélène Bukuru, Thierry Nahimana, Martin Manirakiza,Centre de Prise en Charge Ambulatoire et Multidisciplinaire des PVVIH/SIDA du Centre Hospitalo-Universitaire de Kamenge (CPAMP-CHUK), Burundi; Patrice Barasukana, Hélène Bukuru, Martin Manirakiza, Zacharie Ndizeye, CHUK/Burundi National University, Burundi; Jérémie Biziragusenyuka, Ella Ange Kazigamwa, Centre de Prise en Charge Ambulatoire et Multidisciplinaire des PVVIH/SIDA de l’Hôpital Prince Régent Charles (CPAMP-HPRC), Burundi; Caroline Akoko, Ernestine Kesah, Esther Neba, Denis Nsame, Vera Veyieeneneng, Bamenda Regional Hospital, Cameroon; Bazil Ageh Ajeh, Rogers Ajeh, Dan Ebai Ashu, Eta Atangba, Christelle Tayomnou Deussom, Peter Vanes Ebasone, Ernestine Kendowo, Clarisse Lengouh, Gabriel Mabou, Sandra Mimou Mbunguet, Judith Nasah, Nicoline Ndiforkwah, Marc Lionel Ngamani, Eric Ngassam, George Njie Ngeke, Clenise Ngwa, Anyangwa Sidonie, Clinical Research Education and Consultancy (CRENC), Cameroon; Anastase Dzudie, CRENC and Douala General Hospital, Cameroon; Djenabou Amadou, Joseph Mendimi Nkodo, Eric, Pefura Yone, Jamot Hospital, Cameroon; Annereke Nyenti, Phyllis Fon, Mercy Ndobe, Priscilia Enow, Limbe Regional Hospital, Cameroon; Catherine Akele, Akili Clever, Faustin Kitetele, Patricia Lelo, Kalembelembe Pediatric Hospital, Democratic Republic of Congo; Nana Mbonze, Guy Koba, Martine Tabala, Cherubin Ekembe, Didine Kaba, Kinshasa School of Public Health, Democratic Republic of Congo; Jean Paul Nzungani, Simon Kombela, Dany Lukeba, Sangos plus/Bomoi, Democratic Republic of Congo; Mattieu Musiku, Clement Kabambayi, Job Nsoki,Hopital de Kabinda, Democratic Republic of Congo; Merlin Diafouka, Martin Herbas Ekat, Dominique Mahambou Nsonde, CTA Brazzaville, Republic of Congo; Ursula Koukha, Adolphe Mafoua, Massamba Ndala Christ, CTA Pointe-Noire, Republic of Congo; Jules Igirimbabazi, Nicole Ayinkamiye, Bethsaida Health Center, Rwanda; Providance Uwineza, Emmanuel Ndamijimana, Busanza Health Center, Rwanda; Jean Marie Vianney Barinda, Marie Louise Nyiraneza, Gahanga Health Center, Rwanda; Marie Louise Nyiransabimana, Liliane Tuyisenge, Gikondo Health Center, Rwanda; Catherine Kankindi, Christian Shyaka, Kabuga Health Center, Rwanda; Bonheur Uwakijijwe, Marie Grace Ingabire, Kicukiro Health Center, Rwanda; Beltirde Uwamariya, Jules Ndumuhire, Masaka Health Center, Rwanda; Gerard Bunani, Fred Muyango, Nyagasambu Health Center, Rwanda; Yvette Ndoli, Oliver Uwamahoro, Nyarugunga Health Center, Rwanda; Eugenie Mukashyaka, Rosine Feza, Shyorongi Health Center, Rwanda; Chantal Benekigeri, Jacqueline Musaninyange, WE-ACTx for Hope Clinic, Rwanda; Josephine Gasana, Charles Ingabire, Jocelyne Ingabire, Faustin Kanyabwisha, Gallican Kubwimana, Fabiola Mabano, Jean Paul Mivumbi, Benjamin Muhoza, Athanase Munyaneza, Gad Murenzi, Francoise Musabyimana, Allelluia Giovanni Ndabakuranye, Fabienne Shumbusho, Patrick Tuyisenge, Francine Umwiza, Research for Development (RD Rwanda) and Rwanda Military Hospital, Rwanda; Jules Kabahizi, Janviere Mutamuliza, Boniface Nsengiyumva, Ephrem Rurangwa, Rwanda Military Hospital, Rwanda; Eric Remera, Gallican Nshogoza Rwibasira, Rwanda Biomedical Center, Rwanda.

## Coordinating and Data Centers:

Adebola Adedimeji, Kathryn Anastos, Lynn Murchison, Viraj Patel, Jonathan Ross, Marcel Yotebieng, Natalie Zotova, Albert Einstein College of Medicine, USA; Ellen Brazier, Zachary Dietrich, Heidi Jones, Elizabeth Kelvin, Denis Nash, Saba Qasmieh, Matthew Romo, Olga Tymejczyk, Institute for Implementation Science in Population Health, Graduate School of Public Health and Health Policy, City University of New York (CUNY), USA; Batya Elul, Columbia University, USA; Xiatao Cai, Don Hoover, Hae-Young Kim, Chunshan Li, Qiuhu Shi, Data Solutions, USA; Kathryn Lancaster, The Ohio State University, USA; Mark Kuniholm, University at Albany, State University of New York, USA; Andrew Edmonds, Angela Parcesepe, Jess Edwards, University of North Carolina at Chapel Hill, USA; Olivia Keiser, University of Geneva; Stephany Duda; Vanderbilt University School of Medicine, USA; April Kimmel, Virginia Commonwealth University School of Medicine, USA.

# East Africa IeDEA

## Site investigators and cohorts

Diero L, Ayaya S, Sang E, MOI University, AMPATH Plus, Eldoret, Kenya; Bukusi E, Edwin Mulwa, George Nyanaro, KEMRI (Kenya Medical Research Institute), Kisumu, Kenya; Charles Kasozi, Mathew Ssemakadde, Masaka Regional Referral Hospital, Masaka, Uganda; Winnie Muyindike, Helen Byakwaga, Michael Kanyesigye, Mbarara University of Science and Technology (MUST), Mbarara, Uganda; Barbara Castelnuovo, Aggrey Semeere, John Michael Matovu, Infectious Diseases Institute (IDI), Mulago, Uganda; Fred Nalugoda, Francis X. Wasswa, Rakai Health Sciences Program, Kalisizo, Uganda; Paul Kazyoba, Mary Mayige, (NIMR), Dar es Salaam, Tanzania; Rita Elias Lyamuya, Francis Mayanga, Morogoro Regional Hospital, Morogoro, Tanzania; Kapella Ngonyani, Jerome Lwali, Tumbi Regional Hospital, Pwani, Tanzania; Mark Urassa, Charles Nyaga, Richard Machemba, National Institute for Medical Research (NIMR), Kisesa HDSS, Mwanza, Tanzania; Kara Wools-Kaloustian, Constantin Yiannoutsos, Beverly Musick, Indiana University School of Medicine, Indiana University, Indianapolis, IN, USA; Batya Elul, Columbia University, New York City, NY, USA; Neelima Navuluri, Duke University, Durham, NC, USA; Rachel Vreeman, Mt. Sinai, New York, USA; Jeffrey Martin, Megan Wenger, Craig Cohen, Jayne Kulzer, University of California, San Francisco, CA, USA; Rena Patel, University of Washington, Seattle, WA, USA

# NA-ACCORD

## NA-ACCORD Collaborating Cohorts and Representatives:

AIDS Clinical Trials Group Longitudinal Linked Randomized Trials: Constance A. Benson and Ronald J. Bosch; AIDS Link to the IntraVenous Experience: Gregory D. Kirk; Emory- Grady HIV Clinical Cohort: Vincent Marconi and Jonathan Colasanti; Fenway Health HIV Cohort: Kenneth H. Mayer and Chris Grasso; HAART Observational Medical Evaluation and Research: Robert S. Hogg, Viviane Lima, P. Richard Harrigan, Julio SG Montaner, Benita Yip, Julia Zhu, and Kate Salters; HIV Outpatient Study: Kate Buchacz and Jun Li; HIV Research Network: Kelly A. Gebo and Richard D. Moore; Johns Hopkins HIV Clinical Cohort: Richard D. Moore; John T. Carey Special Immunology Unit Patient Care and Research Database, Case Western Reserve University: Jeffrey Jacobson; Kaiser Permanente Mid-Atlantic States: Michael A. Horberg; Kaiser Permanente Northern California: Michael J. Silverberg; Longitudinal Study of Ocular Complications of AIDS: Jennifer E. Thorne; MACS/WIHS Combined Cohort Study: Todd Brown, Phyllis Tien, and Gypsyamber D’Souza; Maple Leaf Medical Clinic: Graham Smith, Mona Loutfy, and Meenakshi Gupta; The McGill University Health Centre, Chronic Viral Illness Service Cohort: Marina B. Klein; Multicenter Hemophilia Cohort Study–II: Charles Rabkin; Ontario HIV Treatment Network Cohort Study: Abigail Kroch, Ann Burchell, Adrian Betts, and Joanne Lindsay; Parkland/UT Southwestern Cohort: Ank Nijhawan; Retrovirus Research Center, Universidad Central del Caribe, Bayamon Puerto Rico: Robert F. Hunter-Mellado and Angel M. Mayor; Southern Alberta Clinic Cohort: M. John Gill; Study of the Consequences of the Protease Inhibitor Era: Jeffrey N. Martin; Study to Understand the Natural History of HIV/AIDS in the Era of Effective Therapy: Jun Li and John T. Brooks; University of Alabama at Birmingham 1917 Clinic Cohort: Michael S. Saag, Michael J. Mugavero, and James Willig; University of California at San Diego: Laura Bamford and Maile Karris; University of North Carolina at Chapel Hill HIV Clinic Cohort: Joseph J. Eron and Sonia Napravnik; University of Washington HIV Cohort: Mari M. Kitahata and Heidi M. Crane; Vanderbilt Comprehensive Care Clinic HIV Cohort: Timothy R. Sterling, David Haas, Peter Rebeiro, and Megan Turner; Veterans Aging Cohort Study: Lesley Park and Amy Justice

## NA-ACCORD Study Administration:

Executive Committee: Richard D. Moore, Keri N. Althoff, Stephen J. Gange, Mari M. Kitahata, Jennifer S. Lee, Michael S. Saag, Michael A. Horberg, Marina B. Klein, Rosemary

G. McKaig, and Aimee M. Freeman; Administrative Core: Richard D. Moore, Keri N. Althoff, and Aimee M. Freeman; Data Management Core: Mari M. Kitahata, Stephen E. Van Rompaey, Heidi M. Crane, Liz Morton, Justin McReynolds, and William B. Lober; Epidemiology and Biostatistics Core: Stephen J. Gange, Jennifer S. Lee, Brenna Hogan, Bin You, Elizabeth Humes, Lucas Gerace, Cameron Stewart, and Sally Coburn

**IeDEA Southern Africa**

## Site investigators and cohorts:

Gary Maartens, Aid for AIDS, South Africa; Carolyn Bolton, Centre for Infectious Disease Research in Zambia (CIDRZ), Zambia; Robin Wood, Gugulethu (Desmond Tutu HIV Centre), South Africa; Nosisa Sipambo, Harriet Shezi Children’s Clinic, South Africa; Frank Tanser, Hlabisa (Africa Health Research Institute), South Africa; Andrew Boulle, Khayelitsha ART Programme, South Africa; Geoffrey Fatti, Kheth’Impilo AIDS Free Living, South Africa; Safari Mbewe, Lighthouse Trust, Malawi; Elvira Singh, National Cancer Registry (National Health Laboratory Service), South Africa; Cleophas Chimbetete, Newlands Clinic (Ruedi Luethy Foundation Zimbabwe), Zimbabwe; Karl Technau, Rahima Moosa Mother and Child Hospital, South Africa; Brian Eley, Red Cross War Memorial Children’s Hospital, South Africa; Josephine Muhairwe, SolidarMed Lesotho; Idivino Rafael, SolidarMed Mozambique; Cordelia Kunzekwenyika, SolidarMed Zimbabwe, Matthew P Fox, Themba Lethu Clinic, South Africa; Hans Prozesky, Tygerberg Hospital, South Africa; Andrew Boule, Western Cape Provincial Health Data Centre.

## Data centers:

Marie Ballif, Cam Ha Dao Ostinelli, Matthias Egger, Lukas Fenner, Andreas Haas, Stefanie Hossmann, Radoslaw Panczak, Eliane Rohner, Julien Riou, Tiana Schwab, Veronika W Skrivankova, Lilian Smith, Katayoun Taghavi, Per von Groote, Gilles Wandeler, Anja Wettstein, Elizabeth Zaniewski, Kathrin Zürcher, Institute of Social and Preventive Medicine, University of Bern, Switzerland; Nina Anderegg, Kim Anderson, Andrew Boulle, Chido Chinogurei, Morna Cornell, Mary-Ann Davies, Leigh Johnson, Reshma Kassanjee, Kathleen Kehoe, Mmamapudi Kubjane, Nicola Maxwell, Haroon Moolla, Carl Morrow, Patience Nyakato, Gem Patten, Mpho Tlali, Renee de Waal, Wendy Wiemers, School of Public Health and Family Medicine, University of Cape Town, South Africa.

# IeDEA West Africa

## Site investigators and cohorts

Adult cohorts: Marcel Djimon Zannou, CNHU, Cotonou, Benin; Armel Poda, CHU Souro Sanou, Bobo Dioulasso, Burkina Faso; Oliver Ezechi, National Institute of Medical Research (NIMR), Lagos, Nigeria. Eugene Messou, ACONDA CePReF, Abidjan, Cote d’Ivoire; Henri Chenal, CIRBA, Abidjan, Cote d’Ivoire; Kla Albert Minga, CMSDS, Abidjan, Cote d’Ivoire; Aristophane Tanon, CHU Treichville, Cote d’Ivoire; Moussa Seydi, CHNU de Fann, Dakar, Senegal; Ephrem Mensah, Clinique EVT, Lomé, Togo.

Pediatric cohorts: Caroline Yonaba, CHU Yalgado Ouadraogo; Lehila Bagnan, CNHU, Cotonou, Benin; Jocelyn Dame, Lorna Renner Korle Bu Hospital, Accra, Ghana; Sylvie Marie N’Gbeche, ACONDA CePReF, Abidjan, Ivory Coast; Kouadio Kouakou, CIRBA, Abidjan, Cote d’Ivoire; Madeleine Amorissani Folquet, CHU de Cocody, Abidjan, Cote d’Ivoire; François Tanoh Eboua, CHU de Yopougon, Abidjan, Cote d’Ivoire; Fatoumata Dicko Traore, Hopital Gabriel Toure, Bamako, Mali; Oliver Ezchechi,, Agatha David, Rosemary Audu, NIMR, Lagos, Nigeria; Elom Takassi, CHU Sylvanus Olympio, Lomé,Togo.

## Regional coordination

Antoine Jaquet (PI), Didier Koumavi Ekouevi (PI), François Dabis, Renaud Becquet, Charlotte Bernard, Karen Malateste, Olivier Marcy, Marie Kerbie Plaisy, Elodie Rabourdin, Thierry Tiendrebeogo. ADERA, University of Bordeaux, National Institute for Health and Medical Research (Inserm) UMR1219, Research Institute for Sustainable Development (IRD) EMR 271, Bordeaux Population Health Centre, Bordeaux, France; Désiré Dahourou, Sophie Desmonde, Julie Jesson, Valeriane Leroy. CERPOP, Inserm UMR1295, Toulouse, France; Raoul Moh, Jean-Claude Azani, Kadidja Diarra, Jean Jacques Koffi, Maika Bengali, Abdoulaye Cissé, Guy Gnepa, Eric Komena, Apollinaire Horo, Séverin Lenaud, Simon Boni, Eulalie Kangah, Corinne Moh, Jeanne Eliam, PAC-CI program, CHU Treichville, Abidjan, Côte d’Ivoire.

## Partner institutions

Emory University: Igho Ofotokun (PI), Anandi Sheth, Cecile Delille Lahiri, Chris Martin Washington University: Noëlle Benzekri, Geoffrey Gottlieb, Geneva University: Olivia Keiser
